# Selectivity Control of Cu Nanocrystals in a Gas-Fed Flow Cell through CO_2_ Pulsed Electroreduction

**DOI:** 10.1021/jacs.1c03443

**Published:** 2021-05-06

**Authors:** Hyo Sang Jeon, Janis Timoshenko, Clara Rettenmaier, Antonia Herzog, Aram Yoon, See Wee Chee, Sebastian Oener, Uta Hejral, Felix T. Haase, Beatriz Roldan Cuenya

**Affiliations:** Department of Interface Science, Fritz-Haber Institute of the Max-Planck Society, 14195 Berlin, Germany

## Abstract

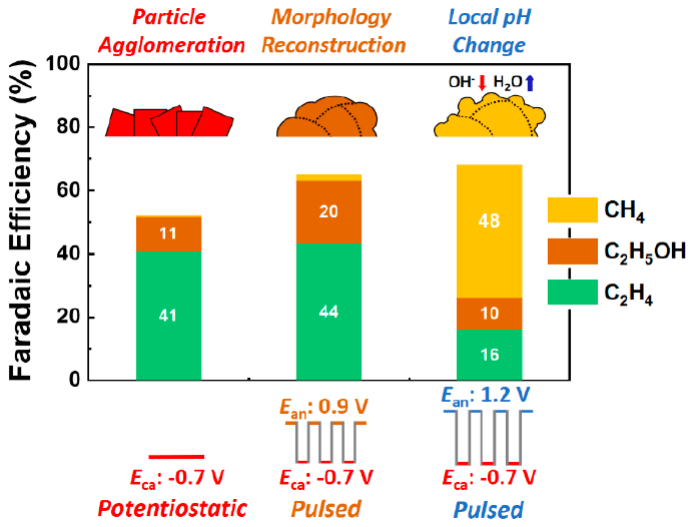

In this study, we have taken advantage of a pulsed CO_2_ electroreduction reaction (CO_2_RR) approach to tune the product distribution at industrially relevant current densities in a gas-fed flow cell. We compared the CO_2_RR selectivity of Cu catalysts subjected to either potentiostatic conditions (fixed applied potential of −0.7 V_RHE_) or pulsed electrolysis conditions (1 s pulses at oxidative potentials ranging from *E*_an_ = 0.6 to 1.5 V_RHE_, followed by 1 s pulses at −0.7 V_RHE_) and identified the main parameters responsible for the enhanced product selectivity observed in the latter case. Herein, two distinct regimes were observed: (i) for *E*_an_ = 0.9 V_RHE_ we obtained 10% enhanced C_2_ product selectivity (FE_C_2_H_4__ = 43.6% and FE_C_2_H_5_OH_ = 19.8%) in comparison to the potentiostatic CO_2_RR at −0.7 V_RHE_ (FE_C_2_H_4__ = 40.9% and FE_C_2_H_5_OH_ = 11%), (ii) while for *E*_an_ = 1.2 V_RHE_, high CH_4_ selectivity (FE_CH_4__ = 48.3% vs 0.1% at constant −0.7 V_RHE_) was observed. *Operando* spectroscopy (XAS, SERS) and *ex situ* microscopy (SEM and TEM) measurements revealed that these differences in catalyst selectivity can be ascribed to structural modifications and local pH effects. The morphological reconstruction of the catalyst observed after pulsed electrolysis with *E*_an_ = 0.9 V_RHE_, including the presence of highly defective interfaces and grain boundaries, was found to play a key role in the enhancement of the C_2_ product formation. In turn, pulsed electrolysis with *E*_an_ = 1.2 V_RHE_ caused the consumption of OH^–^ species near the catalyst surface, leading to an OH-poor environment favorable for CH_4_ production.

## 1 Introduction

The electrochemical CO_2_ reduction (CO_2_RR) driven by electrical energy from renewable sources has attracted attention as an environmentally friendly path to convert the undesired greenhouse gas into feedstock chemicals and fuels.^1,2^ Among the metal catalysts used for the CO_2_RR, copper-based catalysts are of particular interest due to their unique capability to transform CO_2_ into various hydrocarbons and alcohols with high energy density such as CH_4_, C_2_H_4_, and C_2_H_5_OH.^3,4^ However, controlling the selectivity toward a specific product remains the main challenge in this field. Different approaches to address this issue have been explored, such as controlling the morphology of the catalyst and exposed facets,^5−9^ tuning the electrolyte composition,^10,11^ and incorporating secondary metals.^12−17^

Apart from these strategies, applying periodic oxidative potentials during the CO_2_RR was also shown to be an efficient way to steer the selectivity of Cu catalysts toward certain desired products.^18−26^ By choosing the proper values of cathodic and anodic potentials and pulse lengths, the production of CO, CH_4_, C_2_H_4_, and C_2_H_4_OH_5_ could be enhanced. For example, experiments with millisecond pulses have shown that the selectivity of a polycrystalline Cu foil toward syngas (H_2_ and CO) can be improved, which was attributed to a modification of the Cu surface morphology by successive oxidation and reduction.^20^ In another study employing a Cu(100) single crystal, pulsed electrolysis was applied to periodically regenerate Cu(I) species, resulting in a significantly enhanced selectivity for ethanol when highly defective Cu(I)/Cu(0) interfaces were available.^21^ Several other studies have also reported the generation of unusual products such as methanol during the pulsed CO_2_RR, with a concomitant increase in the C_2+_ selectivity (e.g. C_2_H_4_, C_2_H_6_, and propanol), in comparison to that achieved under potentiostatic electrolysis conditions.^22−25^ In the former examples, the selectivity trends observed in H-type cells at low current densities were assigned to the modification of the local chemical environment and concentrations of *OH and *CO adsorbates on the Cu surface. These results are supported by theoretical studies showing that pulsed electrolysis causes changes in the local pH and CO_2_ concentration.^26^

Although pulsed electrolysis has shown remarkable results for controlling the catalyst selectivity, the majority of data available originate from experiments conducted in an H-type cell configuration. However, the current density of CO_2_RR in an H-type cell can only reach a few tens of mA/cm^2^ due to the low solubility of CO_2_ in the electrolyte and the resulting mass transfer limitations.^27,28^ In addition, single crystals that were previously discussed are less attractive for commercial applications due to the limited possibilities to scale up the process and the high catalyst cost. In this regard, a gas-fed flow cell configuration with a gas diffusion electrode (GDE) in an alkaline electrolyte provides an attractive alternative for industrial utilization. It features the three-phase boundary of a CO_2_ gas/liquid/solid interface, can achieve high current density (over 200 mA/cm^2^),^27−31^ and allows using nanoparticulate catalysts.^32^ However, the practical feasibility of such a system depends on the gas-fed flow cell characteristics. Harsh experimental conditions associated with high current densities are expected to affect the chemical and physical properties of the catalysts.^27^ Consequently, *operando* studies in the flow cell configuration are required to shed light on the correlation between the pulsed CO_2_RR parameters and the observed selectivity trends.

Previous studies of the pulsed CO_2_RR in H-type cells have reported that oxidative potentials are beneficial for hydrocarbon and alcohol production.^21,23^ Here we extend such studies to a flow cell configuration and high current densities, using highly active shape-selected Cu_2_O nanocube (Cu NC) catalysts. Herein we demonstrate that pulsed electrolysis in a gas-fed flow cell configuration enables efficient control over the product selectivity, allowing us to switch from C_2_ (C_2_H_4_ and C_2_H_5_OH) to C_1_ (CH_4_) product formation by tuning the value of the applied anodic potential (*E*_an_). To this end, in our pulsed protocol we use higher oxidative potential values than those in previous studies. Such high oxidative pulsed potentials can potentially lead to the transient oxidation of CO_2_RR products, which might have discouraged researchers from exploring wider potential ranges. However, here we show that oxidation of C_2_ CO_2_RR products during the pulses is negligible for the chosen potential range (up to ≤1.2 V vs RHE) and pulse duration (1 s) and that the changes in FE observed are likely related to interfacial pH and structural catalyst changes. Cyclic voltammetry, *operando* X-ray absorption spectroscopy (XAS), *operando* surface-enhanced Raman spectroscopy (SERS), *ex situ* scanning electron microscopy (SEM), and *ex situ* transmission electron microscopy (TEM) measurements allowed us to extract correlations between the catalytic properties and the structure and chemical state of the catalyst.

## 2 Experimental Section

### 2.1 Preparation of Cu NCs and Electrodes

Cu NCs were prepared by modifying a previously reported procedure.^32,33^ In a typical synthesis, a dilute alkaline solution containing Cu ions was prepared by adding 8 mL of a CuSO_4_·6H_2_O solution (0.1 M) and 28 mL of a NaOH solution (1 M) to 732 mL of ultrapure water at room temperature. After the mixture was stirred for 10 s, 32 mL of an l-ascorbic acid solution (0.25 M) was added. The solution was then stirred for a further 13 min. The solution was centrifuged and washed several times with water and ethanol.

To prepare the electrodes on a GDE, a catalyst ink was made by dispersing 0.5 mg of the catalyst powder with ∼22 wt % of Nafion (relative to the total loading, Sigma-Aldrich) in 1 mL of methanol. The ink was then ultrasonicated for 30 min. The as-prepared ink was spray-coated on the microporous layer (MPL) of a gas diffusion electrode (GDE, Sigracet 39bb) using an airbrush. The loading was determined by weighing the GDE before and after the spray coating and was found to be roughly 0.25 mg/cm^2^.

### 2.2 Electrochemical Measurements for CO_2_ Reduction Reaction

Electrochemical CO_2_ reduction experiments were performed in a gas-fed flow cell configuration (Figure S1).^34^ The flow cell consisted of three compartments for CO_2_ gas, catholyte, and anolyte. A catalyst deposited on the GDE working electrode was positioned between the CO_2_ gas and the catholyte chamber, with the catalyst side of the GDE facing the electrolyte. The catholyte and anolyte chambers were equipped with a leak-free Ag/AgCl reference electrode (Innovative Instruments) and a platinum-mesh counter-electrode (MaTecK). An anion exchange membrane (Fumasep FAA-PK-130) was mounted between the catholyte and the anolyte chamber. Gaseous CO_2_ was passed behind the gas diffusion layer at a constant flow of 10 sccm by means of a mass flow controller (Bronkhorst). Note that, since a fraction of CO_2_ gas is converted into products, the flow rate at the outlet of the CO_2_ gas chamber was remeasured by a volumetric digital flow meter (Agilinet ADM 1000) and used for the Faradaic efficiency calculations. An aqueous solution of 1 M KOH (pH 13.7, Acros-Organics) was used as an electrolyte, as well as a phosphate buffer (pH 7.4 and pH 6.4, Sigma-Aldrich) and citric acid (pH 5, Sigma-Aldrich) for auxiliary experiments. The electrolytes were circulated in both compartments by a dual-channel peristaltic pump (FAUST PLP380) at a constant flow controlled by the pump’s rotation speed, which was set to 10 rpm. The electrochemical experiments were performed using a potentiostat (Autolab PGSTAT302N). The Ohmic resistance was determined by electrochemical impedance spectroscopy (EIS). The potential values after *iR* compensation were converted to the reversible hydrogen electrode (RHE) reference scale using *E*(vs RHE) = *E*(vs Ag/AgCl) + 0.242 V + 0.059 V × pH – *iR*.

The gas products were quantified by a gas chromatograph (GC, Agilent 7890B) equipped with a thermal conductivity detector (TCD) and a flame ionization detector (FID). The GC was directly connected to the CO_2_ gas chamber of the flow cell for online analysis. The formic acid and acetate concentrations were analyzed by a high-performance liquid chromatograph (HPLC, Shimadzu Prominence) equipped with a NUCLEOGEL SUGAR 810 column and refractive index detector (RID). The alcohol and aldehyde concentrations were quantified with a liquid GC (L-GC, Shimadzu 2010 plus) equipped with a fused silica capillary column and FID detector. The details of Faradaic efficiency calculations are given in Supplementary Note 1 in the Supporting Information.

### 2.3 *Ex Situ* Characterization

The surface morphology and structure of the catalysts were investigated using SEM (Thermo Fisher Scientific, Apreo SEM) and TEM (JEOL Ltd., ARM200F). Samples for TEM were prepared by coating a nickel grid (400 mesh with a lacey-carbon film, PLANO GmbH) with the catalyst dispersed in hexane before and after the CO_2_RR.

### 2.4 *Operando* Characterization

The high surface to volume ratio in these (initially cubic) nanostructured catalysts enhances the contribution of the active surface sites to the signal yielded by sample-averaging spectroscopic techniques. This is instrumental for *operando* tracking the changes in the morphology and chemical state of the catalysts during the CO_2_RR using methods such as XAS.

*Operando* XAS measurements were performed at the P64 beamline of the PETRA-III synchrotron (Hamburg, Germany). Time-resolved quick X-ray absorption fine structure (QXAFS) spectra were collected in fluorescence mode. A homemade flow cell was used to acquire the XAS spectra under the reaction conditions. In the *operando* flow cell, a Kapton window was installed to allow X-rays to illuminate the catalyst on the GDE from the back and collect the emitted fluorescence (Figure S2). All samples were measured in air and under *operando* conditions corresponding to potentiostatic and pulsed electrolysis. The details of the XAS data acquisition and processing, as well as schematics of the flow cell employed, are given in Supplementary Note 2 in the Supporting Information.

*Operando* surface-enhanced Raman spectroscopy (SERS) was carried out with a Raman spectrometer (Renishaw, InVia Reflex) coupled with an optical microscope (Leica Microsystems, DM2500M) together with a motorized stage for sample tracking (Renishaw, MS300 encoded stage). Calibration of the system was performed using a Si(100) wafer (520.5 cm^–1^). A near-infrared laser (Renishaw, RL785, λ = 785 nm, *P*_max_ = 500 mW) served as the excitation source. The backscattered light was Rayleigh-filtered, and the Raman scattering was collected in the range of 100–1200 cm^–1^ with a grating of 1200 lines mm^–1^ and directed to a CCD detector (Renishaw, Centrus). For the *operando* measurements, the excitation source was focused on the surface of the sample and Raman scattering signals were collected with a water immersion objective (Leica microsystems, 63×, numerical aperture 0.9) protected from the electrolyte by a Teflon film (DuPont, film thickness of 0.013 mm) wrapped around the objective. The collection of each spectrum was performed with 5 s of exposure time. The Raman data were processed using the Renishaw WiRE 5.2 software. The spectra were baseline-subtracted using the polynomial feature of eighth order, and cosmic rays were removed.

## 3 Results and Discussion

**Figure 1:**
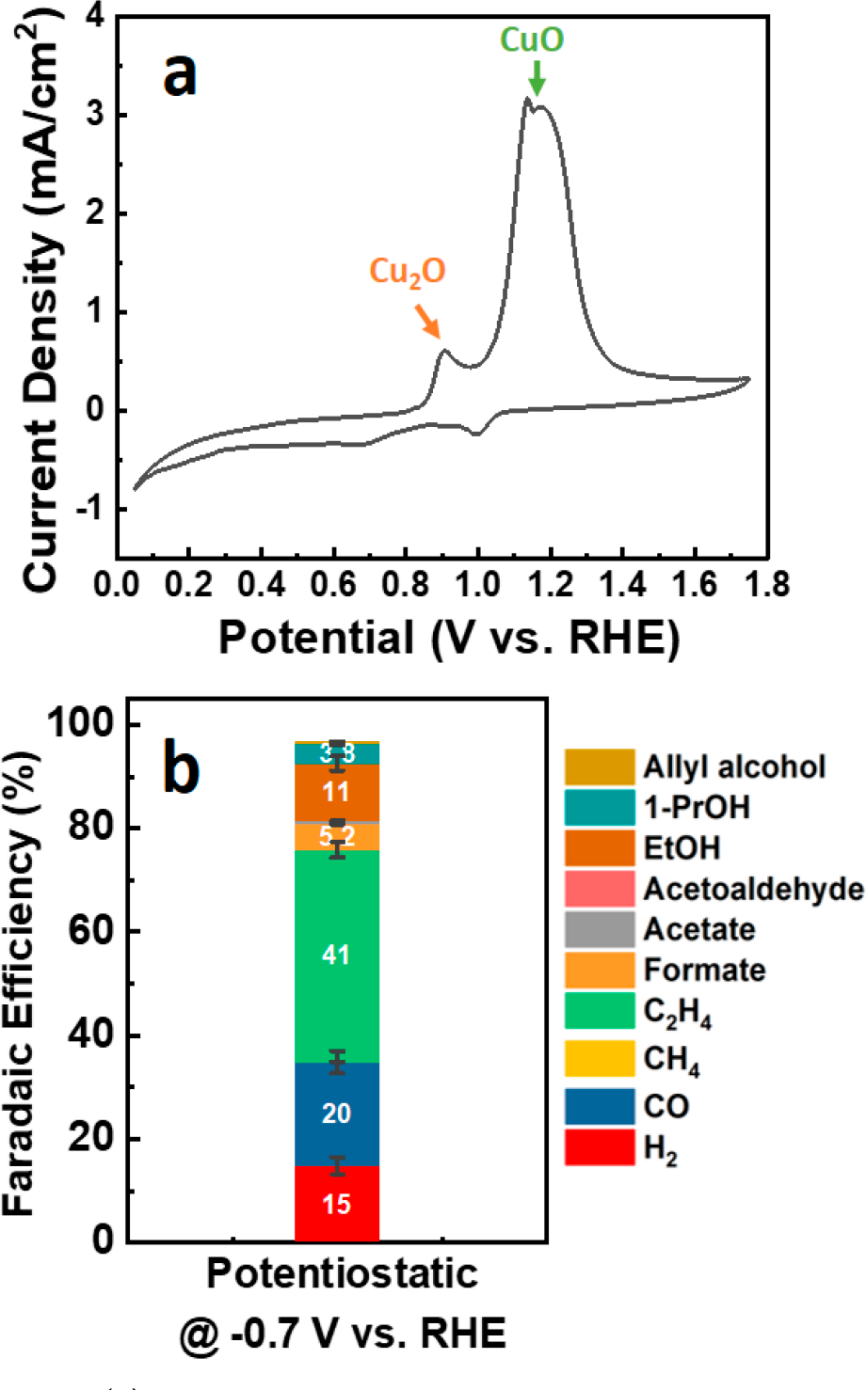
(a) Cyclic voltammogram of the Cu NCs obtained at a scan rate of 1 mV/s in the 1 M KOH electrolyte. (b) FE of Cu NCs under potentiostatic conditions at −0.7 V vs RHE in the flow cell.

We first measured the cyclic voltammograms of Cu NCs in KOH electrolyte to establish the relevant potential values for the pulsed CO_2_RR conditions. As shown in Figure 1a, we observed two distinct anodic peaks at 0.9 and 1.2 V vs RHE in the CV curves. To identify these two oxidation peaks, we tracked the chemical state of the catalyst by means of XAS (see Supplementary Note 3 and Figures S3 and S4). XAS data revealed that these CV peaks can be assigned to the formation of Cu_2_O and CuO, respectively, which is in good agreement with the literature.^35^ We note, however, that according to XAS the formation of Cu(I) species starts at potentials as low as 0.6 V but is significantly faster at higher potentials. Furthermore, oxidation of Cu to Cu(II) state starts at ca. 1.0 V, but even at these high potentials, the formation of new Cu(I) species is still significant and surpasses that of Cu(II) species. We attribute that, in part, to the instability of Cu(II) under the given reaction conditions, which results in leaching of Cu(II), and to the partial re-reduction of Cu(II) back to Cu(I) (see Supplementary Note 3).

On the basis of the CV data we selected the pulsed electrolysis conditions. Specifically, a 1 s pulse at an anodic potential (*E*_an_) was followed by a 1 s pulse at a cathodic potential (*E*_ca_), and the sequence was repeated for 30 min. The *E*_an_ was varied from 0.6 to 1.5 V vs RHE, while the *E*_ca_ value for the CO_2_RR was held at −0.7 V vs RHE. All potentials mentioned in the text are referenced vs the RHE, unless stated otherwise. Next, as a control experiment, the selectivity of the Cu NCs under potentiostatic conditions (at −0.7 V for 30 min) was examined (Figure 1b).

It was observed that the current density under these conditions was −254 mA/cm^2^ and the main products were C_2_ products, in particular, C_2_H_4_ (FE of 40.9%) and C_2_H_5_OH (FE of 11%), which is consistent with previous studies.^32^ This control experiment serves as a standard for comparing and analyzing the upcoming results of the pulsed electrolysis.

**Figure 2:**
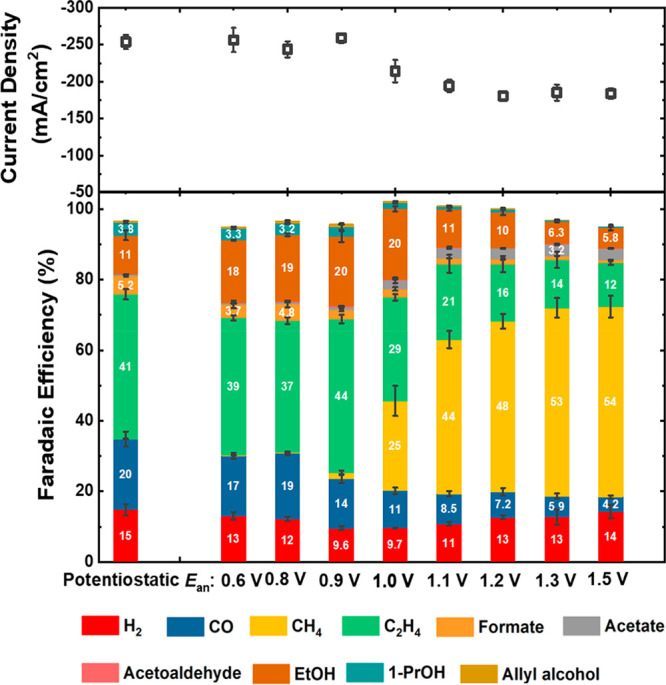
Current density (top) and FE (bottom bar graph) at a potentiostatic −0.7 V vs RHE and under pulsed electrolysis conditions with the different *E*_an_ values indicated and the same *E*_ca_ = −0.7 V vs RHE cathodic potential in all cases. The activity and selectivity data reported are an average of at least three different measurements on analogously prepared fresh independent samples, and the error is estimated as the standard deviation.

The current densities and FE data for the potentiostatic (reference) and pulsed electrolysis with different values of the anodic potential (*E*_an_) are shown in Figure 2. All catalytic activity data were collected after 30 min of the CO_2_RR under the given conditions. High current densities similar to those observed under potentiostatic conditions were also obtained under the pulsed CO_2_RR with an *E*_an_ value of less than 1.0 V. Nevertheless, a further increase in the *E*_an_ value (≥1.0 V vs RHE) results in a remarkable reduction of the current density by ca. 30% in comparison to that under potentiostatic conditions. The FE of the pulsed electrolysis also showed different selectivity trends depending on the *E*_an_ value. The FE at *E*_an_ = 0.6 V showed a selectivity toward C_2_ products similar to that observed under potentiostatic conditions. Nonetheless, upon an increase in the *E*_an_ value to 0.9 V, the C_2_ product selectivity increased and achieved a total C_2_ FE of 63.6% (with a FE for C_2_H_4_ of 44% and a FE for C_2_H_5_OH of 20%), which is about 10% higher than that of the potentiostatic CO_2_RR for the same cathodic potential of −0.7 V. Interestingly, a further increase in the anodic potential does not result in an improved selectivity to C_2_ products. Instead, at *E*_an_ = 1.0 V, the C_2_ product selectivity decreases and the CH_4_ product selectivity is enhanced. At *E*_an_ values over 1.0 V, CH_4_ was the main reaction product and achieved a maximum of 53.9% FE at *E*_an_ = 1.5 V.

The formation of Cu oxide species between ∼0.8 and ∼1.5 V as observed during the CV scan in Figure 1 and the changes in the FE in Figure 2 are closely correlated. However, to accurately characterize the oxidation state of Cu under these reactive conditions, *operando* spectroscopic methods are needed, as shown below.

**Figure 3:**
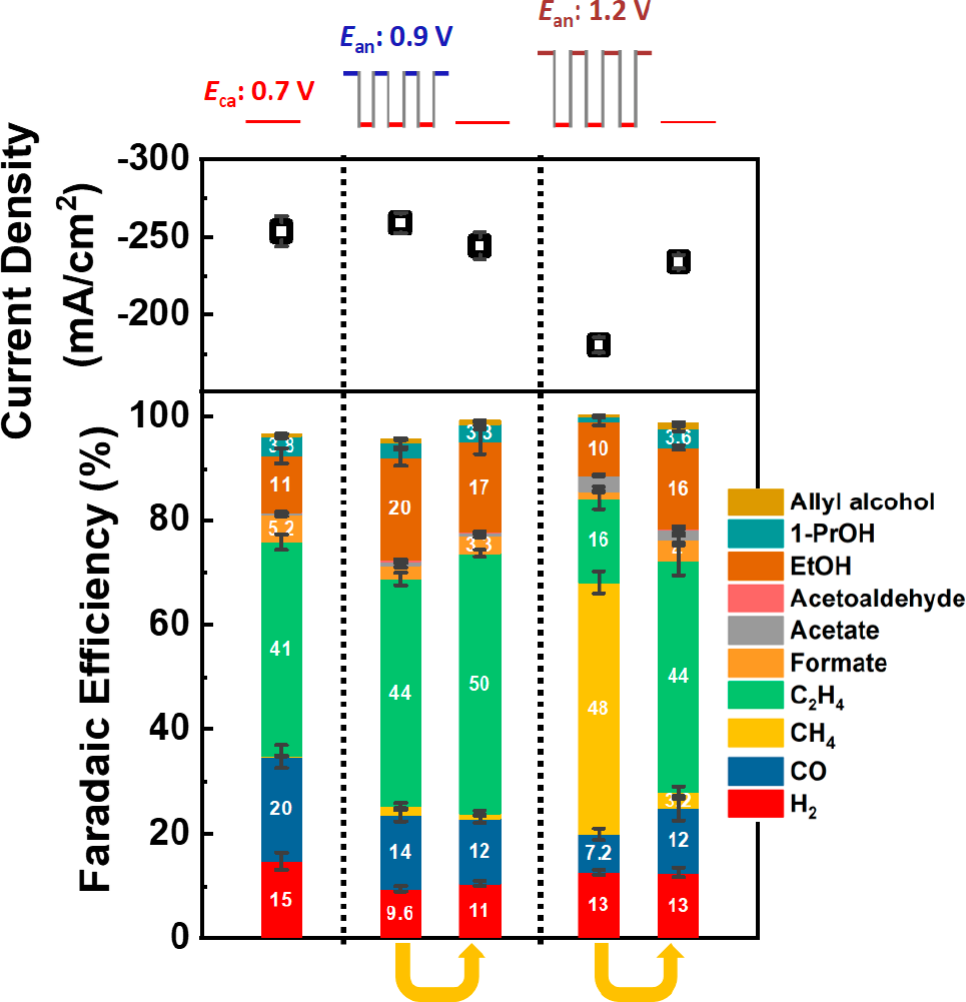
Current density (top) and FE (bottom bar graph) of the pulsed electrolysis with *E*_an_ = 0.9 and 1.2 V and the cathodic pulse *E*_ca_ = −0.7 V. The arrows indicate that the same samples pretreated using pulsed electrolysis were subsequently measured at a constant potential of −0.7 V vs RHE. The activity and selectivity data reported are an average of at least three different measurements on analogously prepared fresh independent samples, and the error is estimated as the standard deviation.

We investigated whether the differences in catalyst activity and selectivity could be attributed to dynamic changes in the catalyst structure and composition under pulsed CO_2_RR conditions or whether they are associated with irreversible changes in the catalyst morphology. To this end, we first ran the pulsed electrolysis with either *E*_an_ = 0.9 or 1.2 V for 30 min and then exposed these samples to potentiostatic conditions at −0.7 V. Interestingly, as shown in Figure 3, the results obtained in these two cases showed a completely different behavior. In the case of the treatment with *E*_an_ = 0.9 V pulses, the enhanced C_2_ selectivity was maintained even after the pulses were interrupted and the same catalytic properties were measured under potentiostatic conditions. This result indicates that the enhancement in C_2_ production induced by the pulsed pretreatment can be assigned to irreversible changes in the catalyst morphology. Nevertheless, in the case of the pretreatment with *E*_an_ = 1.2 V pulses, the CH_4_ selectivity observed under the pulsed electrolysis was suppressed after the pulses were stopped, and C_2_ chemicals reappeared as the main CO_2_RR products. In addition, we observed that the current density, partially suppressed under the *E*_an_ = 1.2 V pulsed conditions, recovered the level attained under potentiostatic electrolysis with a fresh catalyst. This allows us to conclude that the suppression of C_2_ product formation, the decrease in current density, and the enhancement in CH_4_ selectivity under pulsed CO_2_RR with *E*_an_ values above 1.0 V are all the results of dynamic and reversible changes induced by the pulsed reaction conditions.

**Figure 4:**
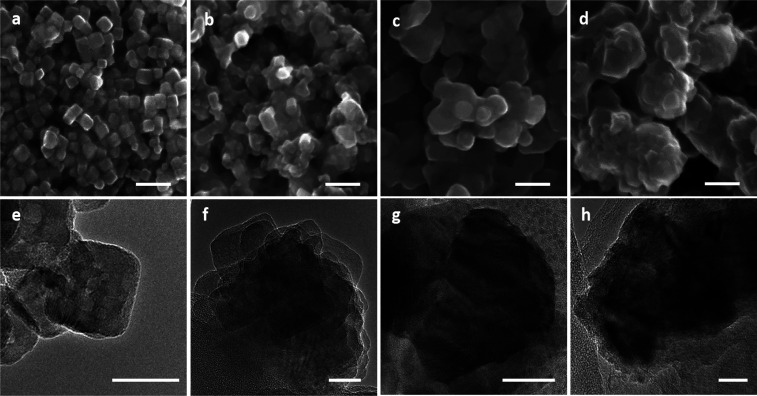
(a–d) SEM and (e–h) TEM images of Cu NCs samples (a, e) before and (b,f) after potentiostatic electrolysis and pulsed CO_2_RR conditions with (c, g) *E*_an_ = 0.9 V and (d, h) 1.2 V. Scale bars: (a–d) 100 nm; (e–h) 20 nm.

**Figure 5:**
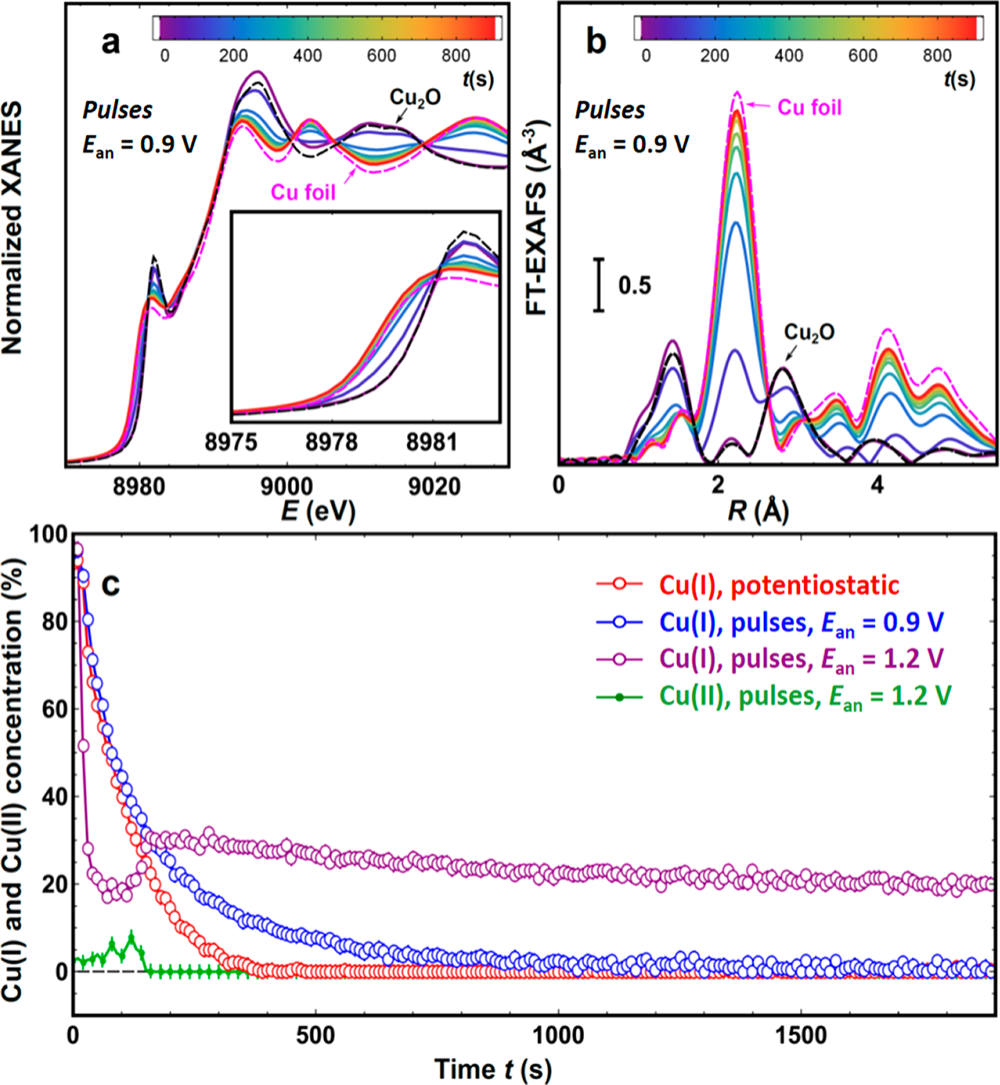
Time-dependent Cu K-edge (a) XANES and (b) Fourier-transformed (FT) EXAFS spectra showing the reduction of Cu NCs under the pulsed CO_2_RR with 1 s pulses and *E*_an_ = 0.9 V. (c) Results of a linear combination fitting of XANES spectra for Cu NCs under the potentiostatic CO_2_RR and pulsed reaction conditions with 1 s pulses and *E*_an_ = 0.9 and 1.2 V. Spectra for metallic Cu, Cu_2_O, CuO, and Cu(OH)_2_ were used as references for the LCA fitting. The Cu(II) concentration reported is the sum of CuO and Cu(OH)_2_ contributions. Weights of the other components are shown in Figure S11.

**Figure 6:**
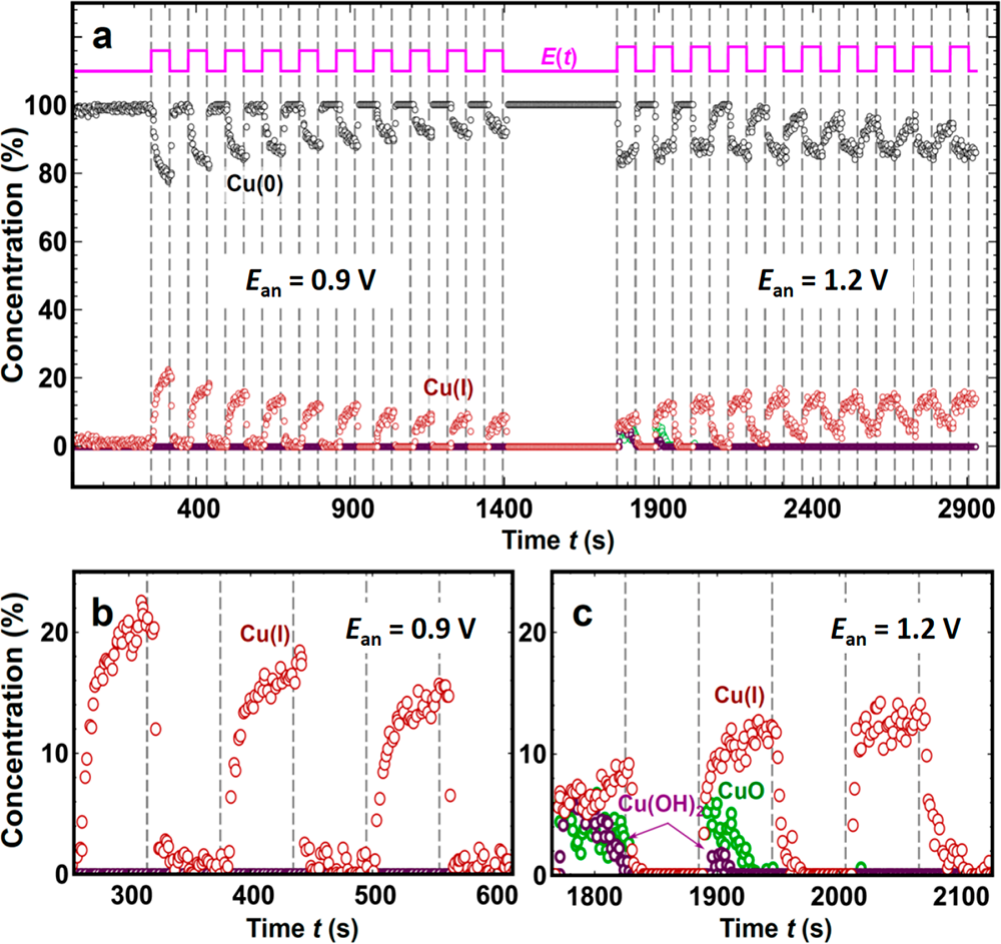
Periodic oxidation and reduction of pre-reduced Cu NCs under 60 s pulses with *E*_ca_ = −0.7 V and *E*_an_ = 0.9 and 1.2 V. (a) Time dependence of the Cu(0), Cu(I), and Cu(II) concentrations, as obtained from LCA-XANES. XANES spectra corresponding to metallic Cu, Cu_2_O, CuO, and Cu(OH)_2_ were used as references. Weights corresponding to CuO and Cu(OH)_2_ are shown separately (green and purple circles, respectively). The sequence of applied potential pulses is also shown in (a). (b, c) Enlarged regions of (a), corresponding to the first three pulses with (b) *E*_an_ = 0.9 V and (c) *E*_an_ = 1.2 V.

We further elucidated the respective role of irreversible changes in the Cu NC morphology and that of dynamic (and reversible) changes in the Cu NC structure on the catalytic properties under pulsed CO_2_RR conditions. For this purpose, we performed *ex situ* SEM and TEM measurements of samples in their as-prepared state and after the potentiostatic and pulsed electrolysis (Figure 4), as well as time-resolved *operando* XAS (Figure 5) and *operando* SERS measurements (Figure 6).

Regardless of the electrolysis mode, the SEM images showed that the well-defined cubic shape (31 ± 4 nm in size) of the as-prepared samples changed after the reaction. In particular, the samples exposed to the pulsed electrolysis evolved into irregularly shaped agglomerates larger than those found in the samples treated under potentiostatic CO_2_RR. The TEM images illustrate more clearly the morphological differences among the three sample types after the reaction. The sample exposed to potentiostatic CO_2_RR conditions transformed into agglomerates that have Cu NCs stacked on top of each other. The agglomerated Cu NCs also partially preserved their cubic shapes. This shape stability is consistent with observations by Buonsanti et al.,^36^ who reasoned that maintaining the particle shape after CO_2_RR is a consequence of the lower potentials required in the gas-fed flow cell (ca. −0.7 V vs RHE) in comparison to the H-type cell configuration, where typically −1.0 to −1.1 V vs RHE is used.

On the other hand, the sample after pulsed electrolysis with *E*_an_ = 0.9 V did not show a structure consisting of agglomerated cubes. Instead, the NCs transformed into larger particles. At *E*_an_ = 1.2 V, we also observed Cu_2_O particles on the surface of the Cu catalysts in some cases (Figure S5), but this cannot be unambiguously attributed to the effect of pulsed electrolysis because the samples were transferred in air. Nevertheless, it is clear that the Cu reconstruction under pulsed conditions was more significant in comparison to the potentiostatic treatment and led to the creation of catalyst particles with a rougher surface. Figure S6 compares scanning TEM and energy dispersive X-ray spectroscopy (EDX) maps of the samples after treatment with potentiostatic and pulsed electrolysis conditions with *E*_an_ = 0.9 and 1.2 V, respectively. The maps suggest a higher oxygen content in the pulsed samples, but we were not able to collect higher signal-to-noise maps due to the presence of the Nafion binder.

*Operando* XAS provides further details about the changes in the catalyst structure under different CO_2_RR regimes. Changes in the Cu K-edge X-ray absorption near-edge structure (XANES) and Fourier-transformed (FT) extended X-ray absorption fine structures (EXAFS) spectra for fresh Cu NCs exposed to pulsed reaction conditions with *E*_an_ = 0.9 V are shown in Figure 5a,b. Raw EXAFS data and the corresponding plots for the potentiostatic CO_2_RR and pulsed reaction conditions with *E*_an_ = 1.2 V are shown in Figure S7 in the Supporting Information. In all cases we observe that Cu, which in the fresh samples is predominantly in the Cu(I) state, is gradually reduced, as indicated by a characteristic shift of the absorption edge to higher energies and a change in the XANES features (Figure 5a), as well as a decrease in the peaks in the FT-EXAFS spectra at ca. 1.5 and 3 Å (phase uncorrected) that correspond to the Cu_2_O-like phase. At the same time, a new peak in the FT-EXAFS develops at ca. 2 Å (phase uncorrected), corresponding to Cu–Cu bonds in metallic Cu. In order to extract quantitative information, we performed linear combination analysis (LCA) of the XANES spectra (Figure 5c), as well as fitting of the EXAFS data (Figures S8–S10).

As shown in Figure S11, both approaches provide good agreement with respect to the changes in the concentrations of metallic and oxidized Cu species. The small discrepancies observed in some cases in the Cu(0) content are attributed to disorder effects in the EXAFS data.

We observe that the transformations in the catalyst’s oxidation state under potentiostatic CO_2_RR conditions and in the pulsed regime with *E*_an_ = 0.9 V proceed in a qualitatively similar way, although the reduction is slower in the latter case. Note here that the time resolution of our XAS experiment (ca. 5 s per spectrum in this case) is not sufficient to track the changes in the catalyst oxidation state during the individual 1 s potential pulses. Nevertheless, the average oxidation states of the Cu NCs after ca. 1500 s of potentiostatic CO_2_RR conditions at −0.7 V and under pulsed CO_2_RR (with *E*_an_ = 0.9 V and *E*_ca_ = −0.7 V) are practically the same.

This finding is different from that reported in a recent *operando* XAS study on another oxide-derived catalyst under pulsed reaction conditions, where an increase in the average catalyst oxidation state was reported under pulsed conditions with *E*_an_ = 0.5 V.^37^ We attribute this difference to the much shorter oxidative pulse lengths employed in our study (1 s here vs 10 s in ref (37)) and to the much higher current densities achieved in our flow cell setup during the cathodic pulse, resulting in a faster catalyst reduction. Moreover, irreversible changes in the catalyst morphology seem to diminish the reoxidation efficiency of our catalyst. To demonstrate this, in Figure 6 we show the results of a XANES analysis for an additional experiment, where longer pulses (60 s) with the same *E*_an_ and *E*_ca_ values were applied. The increased pulse length allowed us in this case to directly track the time-dependent changes in the catalyst composition and structure using *operando* QXAFS. The results obtained support that the periodic application of the *E*_an_ = 0.9 V potential results in a significant reoxidation of the catalyst (Figure 6a). The cationic Cu species generated, however, are removed as soon as the cathodic potential pulse is applied, and more importantly, the reduction of the Cu NCs is clearly much faster than the oxidation (Figure 6b).

Moreover, the amount of oxide generated during each pulse of the anodic potential decreases for each subsequent pulse. The latter is assigned to the irreversible changes in the catalyst morphology taking place during the pulsed protocols. In particular, our TEM data (Figure 4) revealed an increase in the NC size during the pulse treatments, which was also associated with a decrease in the current density due to the smaller surface area available (Figure S17). This reconstructed NC morphology is also expected to affect the oxidation state. Prior literature reports revealed that differently oriented Cu surfaces display different oxidation kinetics.^38,39^ Our {100} nanocubes are transformed during the pulse electrolysis into rougher morphologies with likely distinct oxidation dynamics. While extrapolation of the results collected with 60 s pulses to the case with 1 s pulses should be done with caution, in both cases we observe very similar trends in the sense that the role of the periodically regenerated oxide species diminishes with time, in line with the irreversible changes previously revealed.

The situation is remarkably different when pulses with a higher *E*_an_ value are applied. First, under 1 s pulses at *E*_an_ = 1.2 V the reduction of the Cu_2_O phase initially present is much faster than that under 1 s pulses with *E*_an_ = 0.9 V. Moreover, unlike the potentiostatic and pulsed CO_2_RR cases with lower *E*_an_ values, for *E*_an_ = 1.2 V the pulsed reaction conditions result in the formation of a small fraction of Cu(II) species (Figure 5c). The Cu(II) species, however, are not stable under these conditions and are either dissolved (see Supplementary Note 3) or converted back to Cu(I) species. Indeed, after ca. 200 s under pulsed CO_2_RR with *E*_an_ = 1.2 V, the contribution of Cu(II) decreases to practically zero, while a significant enhancement in the population of Cu(I) species is present. The concentration of Cu(I) species is ca. 20%, which indicates the formation of a thick oxide layer. The conversion of Cu(I) first to Cu(II) and then back to Cu(I) species is also supported by the EXAFS fitting results, which demonstrate an increase in the Cu–O bond length during the first 100 s under pulsed CO_2_RR conditions and then its decrease back to the original value (Figure S12), in agreement with the difference in Cu–O bond length in CuO and Cu_2_O oxides (1.937 and 1.836 Å, respectively). The QXAFS results collected under 60 s pulses (Figure 6) confirm the trends in the data set with 1 s pulses. In particular, the periodic application of an anodic potential drives oscillations in the Cu(I) concentration, while the contribution of Cu(II) species is detectable only during the first few potential cycles (Figure 6c). Moreover, unlike what is observed in the case of *E*_an_ = 0.9 V, under *E*_an_ = 1.2 V pulses, the average concentration of Cu(I) does not decrease with time.

Instead, in this regime a prolonged exposure to pulsed conditions suppresses the catalyst reduction under a cathodic potential and minimizes the difference between the catalyst reduction and oxidation rates. Indeed, as shown in Figure 6a, the changes in the Cu(0) concentration profile during reductive/oxidative pulses with *E*_an_ = 1.2 V are more symmetrical after 10 cycles in comparison to those during the first few cycles and are also more symmetrical than those observed under pulses with *E*_an_ = 0.9 V.

**Figure 7:**
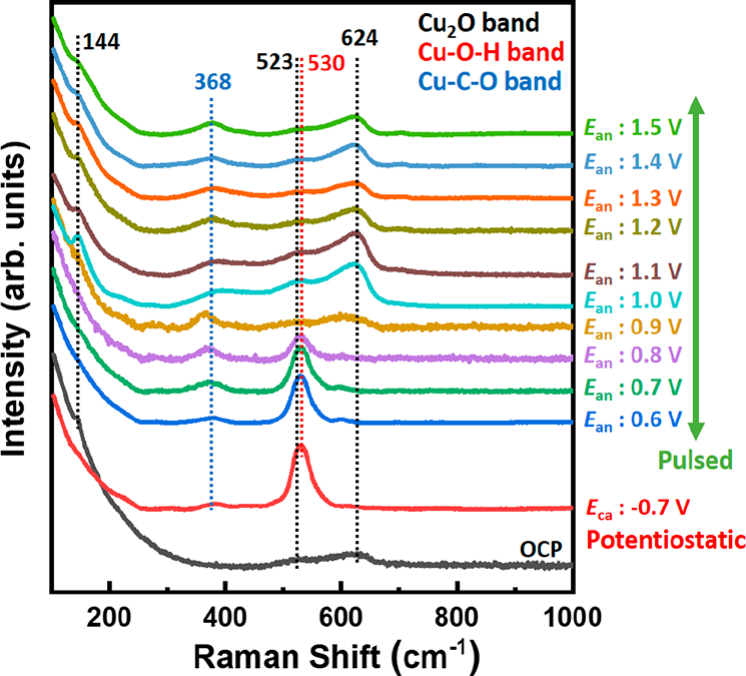
(a) *Operando* surface-enhanced Raman spectra under OCP, potentiostatic operation at −0.7 V, and pulsed conditions with different *E*_an_ values. Dashed lines represent the Raman bands of Cu_2_O (black), Cu–OH (red), and Cu–CO (blue).

Complementing bulk-sensitive XAS data, *operando* SERS measurements provide an important insight into the surface speciation under the CO_2_RR at high current densities and different pulse protocols. Figure 7 shows Raman spectra acquired under an open circuit potential (OCP) and during the potentiostatic and pulsed CO_2_RR. Under the OCP, the characteristic bands of Cu_2_O were observed at 624, 523, and 144 cm^–1^.^40−44^ Under potentiostatic conditions at −0.7 V, these bands vanished. Meanwhile, new bands appeared at 530 and 368 cm^–1^. These bands are associated with the presence of *OH and *CO on the Cu surface, and this finding is thus in line with previous Raman studies carried out for oxide-derived Cu nanostructures in an alkaline electrolyte.^41−43^ Under the pulsed electrolysis conditions, the adsorbed *CO band was detected, regardless of the *E*_an_ value, suggesting that *CO could serve as an intermediate for the production of hydrocarbons. Note here that the time resolution of the SERS measurements (5 s per spectrum) does not allow us to track the changes in the adsorbate coverage during each individual pulse, and therefore it only provides information about the average state of the catalyst surface. In contrast to the results for *CO, the adsorbed *OH band showed a strong dependence on the *E*_an_ value. The pulsed electrolysis treatment with an *E*_an_ value below 0.8 V had no significant influence on the intensity of this band. However, increasing the anodic potential to *E*_an_ = 0.9 V and higher resulted in a noticeable decrease in this band’s intensity, paralleled by the appearance of bands related to the Cu_2_O phase. These Cu_2_O bands were more pronounced at *E*_an_ values over 1.0 V. Therefore, in good agreement with the *operando* XAS data, SERS data also indicated that the pulsed electrolysis at high *E*_an_ values resulted in the accumulation of Cu_2_O species. An important finding from SERS is that the band of absorbed *OH was reduced significantly in intensity when Cu_2_O was accumulated, suggesting that the formation of Cu_2_O consumed the *OH species on the Cu surface. This is a reasonable assumption, considering that in the alkaline electrolyte Cu_2_O is produced under the anodic potential by the reaction of metallic Cu with OH ions (2Cu + 2OH^–^ → Cu_2_O + H_2_O). From this observation, we infer that the local pH near the surface of the Cu NCs must transiently decrease during the pulsed electrolysis.

On the basis of the XAS, SERS, and microscopy results, we conclude that the effect of the pulsed electrolysis in the flow cell configuration on the selectivity for either C_2_ products or CH_4_ depends strongly on the *E*_an_ value. For *E*_an_ values below 0.9 V, the enhanced C_2_ selectivity can be attributed to irreversible changes in catalyst morphology: namely, to the creation of a defective surface with large defective interfaces and grain boundaries under harsh pulsed conditions. These defect sites appear to facilitate the C_2_ product formation. Previous studies in H-type cells have demonstrated that the presence of defects, specific facets, and grain boundaries on the Cu surface promotes the C–C coupling required for C_2_ product generation.^5−8,45−47^ It should be noted not only that the pulse electrolysis treatment employed here affects the catalyst morphology, chemical state, and local pH around the active motifs but also that the oxidative potentials might result in the oxidation of alcohols, aldehyde, and formate to generate more carbonaceous intermediates (including *CO), which are key intermediates to C_2+_ products, particularly C_2_H_4_. However, the latter effect was discarded as a significant contributor to the selectivity trends obtained, since the enhanced C_2+_ selectivity was preserved after the pulse treatment was interrupted and the same sample was subsequently measured under potentiostatic conditions (constant negative applied potential). This indicates that irreversible changes in the sample morphology taking place during the pulse treatment are mainly responsible for the improved C_2+_ selectivity detected.

In the case of pulses with *E*_an_ = 1.2 V, where CH_4_ is produced as the main product, XAS and SERS point to the formation of a Cu_2_O layer that cannot be fully removed during the cathodic pulse. The low conductivity of the Cu_2_O shell formed on the surface of the metallic Cu NCs is likely responsible for the decrease in the current density^48^ observed in Figure 2. Strikingly, this Cu_2_O layer suppresses the formation of C_2_ products yet appears to enhance the formation of CH_4_. This result is intriguing, since normally metallic Cu is considered to be the active site for methane formation. Given that our *operando* SERS data still display the presence of bonds between metallic Cu and *CO adsorbates under pulsed conditions, this result implies that metallic Cu and Cu_2_O coexist on the catalyst surface during pulsed electrolysis. Therefore, we postulate that the active sites for CH_4_ production are still metallic Cu species and that the enhancement in CH_4_ selectivity obtained for *E*_an_ = 1.2 V can be attributed to a pH effect. It is well-known that the CH_4_ production in the CO_2_RR is greatly affected by the local pH.^9,49^ In fact, our *operando* SERS data revealed the consumption of OH species due to the regeneration of Cu_2_O species during the anodic pulse. This condition would lead to local OH consumption, resulting in a rapid decrease in the local pH near the surface of the catalyst. The locally reduced pH may make the reaction pathway favorable toward CH_4_ production. To test this, we used different buffer electrolytes as controls and found that a weakly acidic environment indeed produced CH_4_ as the main product instead of C_2_ products (Figure S13). Interestingly, we also observed that the use of a weak acid as an electrolyte increased the H_2_ selectivity. This observation is different from that for the pulsed electrolysis, where the effective suppression of H_2_ production takes place, which is likely due to the low proton concentration (ca. 10^–13.7^ mol/L) in the bulk KOH electrolyte. Given that H_2_ production is regarded as a competing reaction with CO_2_ reduction, these results highlight that the pulsed electrolysis is an effective way to produce CH_4_, while it suppresses H_2_ production. Furthermore, the complex interactions taking place at the electrolyte–catalyst interface are likely strongly influenced by the presence of the binder/Nafion, which is currently largely overlooked in the literature and requires further study.

The application of high oxidative potentials during the pulse protocol deserves special attention. In general, applying high oxidative potentials can lead to the oxidation of CO_2_RR products, which would strongly affect our aforementioned interpretation based on changes in the local pH. In particular, a decreasing FE for C_2_ products at the expense of an increasing CH_4_ FE could indicate C–C fission during the oxidative pulse. To test this possibility, we studied the oxidation behavior of ethylene and ethanol by recording gas chromatograms during pulsed electrolysis with oxidative pulses ≤1.2 V with our flow cell (Figures S14–S16). For the whole pulse potential range, we did not detect any ethylene or ethanol oxidation products (CO, CH_4_, or CO_2_) above the impurity level in the gaseous stream. Together with the near 100% FE observed in Figure 2 (calculated by only accounting for the cathodic currents), these results strongly suggest that C–C fission does not occur under the conditions tested here, likely because of a high activation energy (for a detailed discussion see Supplementary Note 4).

Finally, stability tests of potentiostatic and pulsed electrolysis were carried out for 10 h (Figures S17 and S18). We observed that the selectivity obtained under the given pulsed conditions was maintained during the reaction. However, it should be noted that the system stability was guaranteed when the electrolyte was periodically refreshed; otherwise, a continuous decrease in the current density was observed during the reaction (Figure S19). The latter is explained by the gradual transformation of KOH to carbonate, resulting in the reduction of the electrolyte conductivity.^50^ Indeed, the change in the selectivity caused by this transformation was more pronounced under the pulsed conditions of CH_4_ production, which is sensitive to the change in the local pH (Figure S17c). For practical commercial applications of the pulsed electrolysis, further work is still required to address this issue, thus ensuring long-term productivity and technoeconomic feasibility. Nonetheless, the pulsed electrolysis appears to be a promising strategy to achieve tunability in the CO_2_RR selectivity at high current densities.

## 4 Conclusion

In summary, we have explored the effect of a pulsed CO_2_ electroreduction procedure on the selectivity of a Cu NC catalyst in a gas-fed flow cell configuration. Our results demonstrate that the hydrocarbon selectivity can be effectively controlled and that one can switch between C_1_ and C_2_ product formation by properly choosing the pulsing conditions. *Operando* spectroscopy and *ex situ* microscopy measurements revealed that such selectivity trends can be assigned to different factors. The irreversible changes in the morphology of the Cu NCs observed after pulsed electrolysis with *E*_an_ = 0.9 V were found to play a key role in the enhancement of the C_2_ product formation. Meanwhile, the OH-poor environment achieved at *E*_an_ = 1.2 V was found to be responsible for the higher selectivity toward CH_4_.

We believe that our findings provide new strategies for controlling the selectivity of Cu catalysts for the electrochemical CO_2_ conversion at high current density and new insight into the fundamental processes governing the catalyst properties in the flow cell configuration. While our study was focused on the effect of the anodic potential (*E*_an_) under the pulsed conditions, we expect that further flexibility in steering the CO_2_ reduction product distribution can be achieved by tuning other parameters of the pulsed protocol employed, such as the cathodic potential (*E*_ca_) and the respective pulse lengths, offering new opportunities for the selective generation of the desired products according to the industrial demand.

## Supporting Information Available

Details of *operando* gas-fed flow cell configurations, XAS data, TEM-EDX data, and electrochemical data (PDF)
