# Supplementary material for: Selectivity Control of Cu Nanocrystals in a Gas-Fed Flow Cell through CO2 Pulsed Electroreduction
Source: J Am Chem Soc. 2021 May 6;143(19):7578–87. doi: 10.1021/jacs.1c03443 (PMC8154520; doi:10.1021/jacs.1c03443)
Supplement: Supplementary file 1 — ja1c03443_si_001.pdf [file ja1c03443_si_001.pdf]

Supplementary Information

Selectivity Control of Cu Nanocrystals in a Gas-Fed Flow Cell through CO_2 Pulsed Electroreduction

Hyo Sang Jeon,[†] Janis Timoshenko,[†] Clara Rettenmaier',[†] Antonia Herzog,[†] Aram Yoon,[†] See Wee Chee,[†] Sebastian Oener,[†] Uta Hejral,[†] Felix T. Haase,[†] and Beatriz Roldan Cuenya*,[†]

[†]Department of Interface Science, Fritz-Haber Institute of the Max-Planck Society, 14195 Berlin, Germany

* Corresponding author. E-mail: roldan@fhi-berlin.mpg.de

Supplementary Note 1: Calculations of the Faradaic efficiency of gas and liquid products. The Faradaic efficiencies (FE) of each product were calculated from the areas of the GC chromatogram as indicated below:

$$i_{partial} = \mathrm{V} \times \text{flow rate} \times \frac{nFp_0}{RT_0} \times \frac{t_{anodic}+t_{cathodic}}{t_{cathodic}} \quad (1)$$

$$FE = \frac{i_{partial}}{i_{total}} \times 100 \quad (2)$$

Here V is the volume concentration of gas products obtained based on a previous calibration of the GC, and the flow rate (mL/min) was measured by a universal flow meter (ADM 1000, Agilent Technologies) at the exit of the flow cell. n is the number of transferred electrons for a certain product, i_{total} (mA) is a steady-state current, F = 96 485 (A s/mol), p_0 = 1.013 (bar), T_0 = 298 (K), and R = 8.314 (J/(mol×K)). t_{anodic} represents the duration of the oxidation phase and t_{cathodic} the duration of the CO_2 electroreduction phase. In this study, in all cases $t_{\mathrm{anodic}} = t_{\mathrm{cathodic}}$ = 1 s.

The Faradaic efficiencies of each product were calculated from the areas of the HPLC or liquid GC chromatogram as indicated below:

$$i_{partial} = \frac{C_{liquid} \times V \times n \times F}{t} \quad (1)$$

$$FE = \frac{i_{partial}}{i_{total}} \times 100 \quad (2)$$

Here C_{liquid} (mol/L) is the concentration of liquid products obtained based on a previous calibration of the HPLC and liquid GC, V (L) is the volume of the electrolyte. n is the number of transferred electrons for a certain product, t (s) is the electrolysis time, i_{total} (mA) is a steady-state current, F = 96 485 ((A×s)/mol).

Supplementary Note 2. XAS experimental details and data analysis

Operando time-resolved X-ray absorption fine-structure spectroscopy (XAFS) experiments were carried out at the Cu K-edge (8979 eV). Measurements were performed in quick XAFS (QXAFS) mode at P64 beamline of PETRA III synchrotron (Germany). A tapered undulator was used as X-ray source, and a Si(111) channel-cut monochromator was used for fast energy selection. The beam-size was less than 2 x 2 mm. The XAS data were collected in fluorescence mode using a PIPS detector, with the rate being between one spectrum per second and one spectrum per 5 seconds. The intensity of the incident radiation was monitored by a gas ionization chamber filled with pure N_2. Additional ionization chambers were used to acquire spectra of a Cu foil in transmission mode for calibration purposes at the beginning of each QXAFS scan.

For *operando* measurements we used a home-made gas-fed electrochemical flow cell, adapted for *operando* experiments. The applied potential was controlled with an *Autolab* potentiostat. As electrolyte we used an aqueous solution of 1 M KOH that was continuously circulated through the cell using a double-channel peristaltic pump. The CO_2 flow in the gas compartment was 10 ml/min.

Data extraction and calibration were performed using the JAQ software of the P64 beamline.[S1] Further data processing, including accurate alignment, atomistic background subtraction, data normalization, spectra averaging and linear combination analysis (LCA) of the X-ray absorption near edge structure (XANES) spectra were performed using a set of *Wolfram Mathematica* scripts.

Extended X-ray absorption fine structure (EXAFS) data extraction and fitting for selected spectra were performed using the *Athena* software and the FEFFIT code.[S2] For EXAFS data modeling (for the 1st coordination shell only), theoretical photoelectron scattering phases and amplitudes were obtained in self-consistent calculations with the FEFF8.5 code[S3] for bulk

reference materials (Cu, Cu_2O, CuO, and $Cu(OH)_2$). The values of the amplitude reduction factors (S_0^2 factors) were obtained in the fitting of EXAFS spectra for bulk reference compounds. In all cases, fitting of the EXAFS spectra $\chi(k)k^2$ was carried out in R-space in the range from R_{min} = 1.1 Å up to R_{max} = 2.8 Å. Fourier transform was carried out in the k range from 2.0 Å^{-1} up to 11.5 Å^{-1}. Fitting parameters were Cu-Cu and Cu-O coordination numbers (N_{Cu-Cu} and $N_{Cu-O,}$ respectively), the corresponding interatomic distances R_{Cu-Cu} and R_{Cu-O} and disorder factors (Debye-Waller or mean-square relative displacement factors) $\sigma^2_{\mathrm{Cu-Cu}}$ and $\sigma^2_{\mathrm{Cu-O}}$. The correction to photoelectron reference energy ΔE_0 was treated as an additional fitting variable.

Supplementary Note 3. Identification of the oxidation potentials for Cu NCs in the flow cell

For the interpretation of the oxidation potentials we collected *in-situ* QXAFS data for Cu nanocubes (NCs) during the cyclic voltammetry (CV) experiment. Before the CVs, the Cu NCs were fully reduced by applying -0.7 V vs. RHE. During the CV, the potential applied to the sample was changed between 0 and + 1.8 V *vs.* RHE, with a rate of 1 mV/s.

Normalized Cu K-edge XANES spectra, acquired during the potential increase from 0 to +1.8 V *vs.* RHE, are shown in **Figure S3a**. By comparing the XANES spectra with those for bulk reference materials we can conclude that the initially reduced Cu NCs are gradually oxidized during the forward scan of the CV. For quantitative analysis we perform LCA, where the experimental normalized Cu K-edge XANES spectra are fitted with a linear combination of standard XANES reference spectra for a Cu foil, Cu_2O, CuO and $Cu(OH)_2$. An example of an LCA fit for a selected potential is shown in **Figure S3b**. The time- and potential-dependencies of the weights of the different contributions to the experimental XANES spectra are shown in **Figure S4**. During the CV, we observed also the decrease in the total Cu fluorescence signal, which is attributed to the partial dissolution of the sample. The time-dependency of the intensity of the fluorescence signal associated with X-ray absorption by copper species is shown in **Figure S4a**.

LCA results summarized in **Figure S4b** show that the metallic state of Cu NCs is preserved until *ca.* 660 s of experiment, where the applied potential reaches *ca.* +0.7 V. A further increase in the applied potential results in a significant oxidation of Cu(0) to Cu(I) species (line A in **Figure S4**). This potential value corresponds to the onset of the 1st anodic peak in the cyclic voltammogram (**Figure 1a** in the main text).

A further increase in the potential to *ca.* 1.0 V leads to the formation of Cu(II) species, as suggested by an increase in the weight corresponding to CuO in the LCA results (line B in

Figure S4). This potential corresponds to the onset of the second anodic peak in **Figure 1a**. However, the accumulation of Cu(II) is not observed, which can be attributed to the instability of these species under the given conditions. Indeed, the onset of the Cu(II) formation coincides with the onset of sample dissolution, as signified by the reduction in the Cu fluorescence signal, shown in **Figure S3a**. Approximately 40% of Cu is lost during the first ten minutes in this regime. Further dissolution of Cu species, however, is significantly slowed down. The dissolution can be mainly attributed to newly formed Cu(II) species, since no significant reduction in the concentration of Cu(I) species was detected. In fact, we observed that the content of Cu(I) species continues to increase, which can be attributed to the reduction of Cu(II) species in contact with the electrolyte back to the Cu(I) state. Note also that no significant dissolution of the catalyst was observed when the applied anodic potential did not exceed the value required for the formation of Cu(II) species.

Generation of Cu(II) species continues as long as the potential exceeds +1.0 V (line D in **Figure S3**). However, we observe that after the CV potential reaches a value of *ca.* +1.7 V (line C), there seems to be a change in the local structure of the species formed, and LCA suggests that Cu(II) species have a local environment now more similar to that of $Cu(OH)_2$ rather than CuO. This can be attributed to significant irreversible changes in the catalyst surface morphology due to the prolonged leaching of Cu(II).

After the potential is decreased below +1.0 V, the remaining Cu(II) species dissolve or get reduced, and enhanced formation of Cu(I) is observed. Formation of Cu(I) continues until the potential falls below 0.4 V (line E in **Figure S4**). The potential corresponding to the Cu(I) reduction to the Cu(0) state thus roughly reflects the position of the broad and shallow cathodic peak in the cyclic voltamogram in **Figure 1a.**

In summary, we conclude that the formation of Cu(I) species is expected at potentials higher than 0.6 V, while the generation of Cu(II) species is less efficient even at higher potentials. Note here also that the lower voltage corresponding to the Cu(I) to Cu(0) transition (line E, 0.4

V) in comparison to that corresponding to Cu(0) to Cu(I) transition (line A, 0.7 V) suggests that irreversible changes in the catalyst structure after exposure to high anodic potential values may decrease its reducibility. This observation will be instrumental for the understanding of the changes in the Cu NCs under pulsed CO_2RR conditions.

Supplementary Note 4: Possible effect of the re-oxidation of carbonaceous species during pulsed electrolysis:

Figure 2 shows the changing FE as a function of the E_{an} value. One possible explanation for the observed change could involve the oxidation of carbonaceous intermediates. In general, applying large oxidative potentials can lead to the oxidation of CO_2RR products. This hypothesis can be however ruled out by a careful consideration of our experimental data.

First, in the case of E_{an} = 0.9 V, where enhanced C_2 product formation is observed, **Figure 3** clearly shows that the C_2 product selectivity was maintained even after the pulses were interrupted and the catalyst selectivity was measured again under potentiostatic conditions. This result indicates that the selectivity observed under these pulsed conditions cannot be attributed to the dynamic processes driven by the oxidative potential pulses, but is rather mainly associated with irreversible changes in the catalyst morphology.

For pulses at E_{an} = 1.2 V, we do not observe pronounced irreversible catalyst changes. Instead, when applying potentiostatic conditions after the pulsed protocol, the initial FE values are regained rapidly. During the application of the pulses at E_{an} = 1.2 V, we observe a decreasing FE for C_2 products at the expense of an increasing CH_4 FE which could indicate C-C fission during the oxidative pulse. To test this possibility, we studied the electro-oxidation behavior of ethylene and ethanol by recording gas chromatograms during pulsed electrolysis with oxidative pulses $\leq$ 1.2V within our flow cell (**Figure S14-S16**). For the whole potential range, we do not detect any ethylene or ethanol oxidation products (CO, CH_4 or CO_2) above the background levels. Furthermore, our total F.E obtained for CO_2 electroreduction (**Figure 2**) was ~100%, calculated only accounting for the cathodic currents. This strongly demonstrates that our products mainly stem from the CO_2 reduction, and that we do not detect any significant fraction of other species arising from the product/intermediate re-oxidation during the pulsed protocols. To be more specific, if re-oxidation processes during the positive potential pulse would be

significant, we would not achieve a total 100% F.E for CO_2RR. In addition, if oxidation reactions during the pulsed electrolysis would be dominant and responsible for the selectivity trends obtained, we should observe their corresponding oxidation currents. However, as can be seen in **Figure S20**, the oxidation current only reached a few mA/cm^2, which was assigned to the formation of Cu_2O species, as evidenced by our *operando* spectroscopy data.

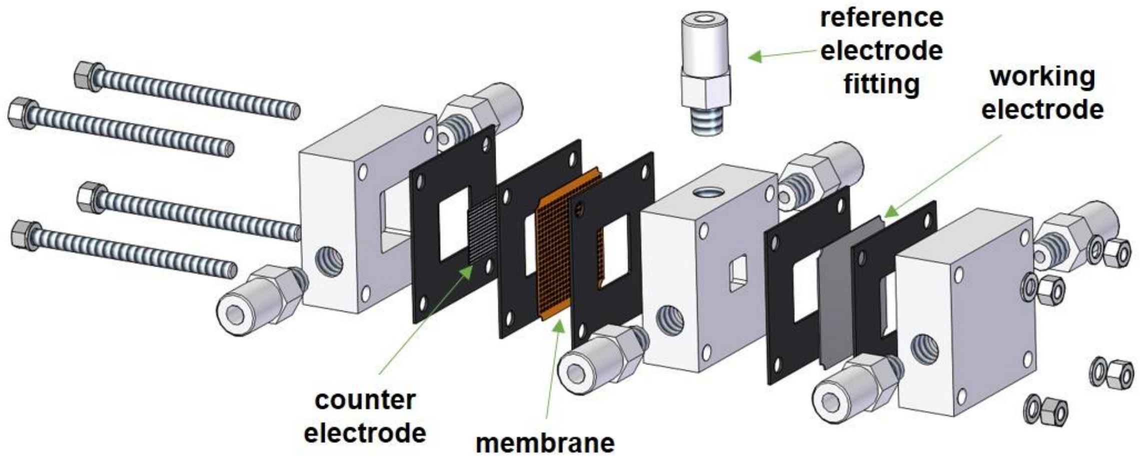

Figure S1. Schematic of the gas-fed flow cell configuration.

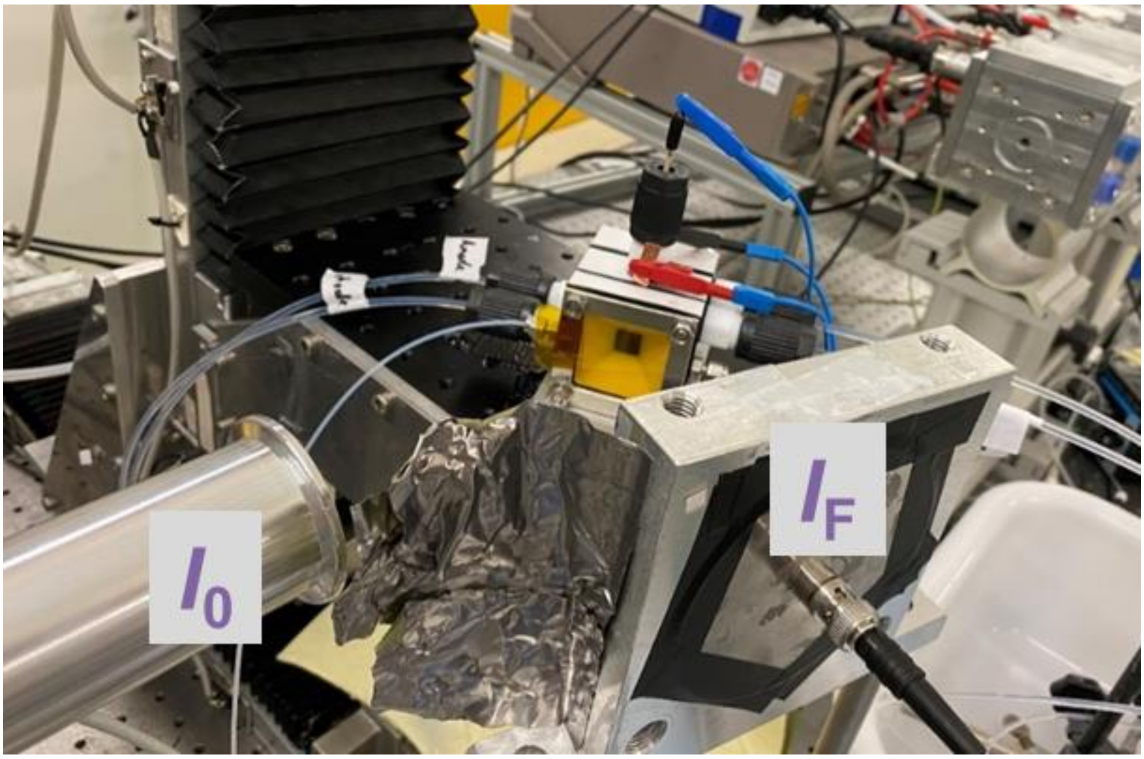

Figure S2. Photograph of the experimental *operando* XAS set-up with the flow cell.

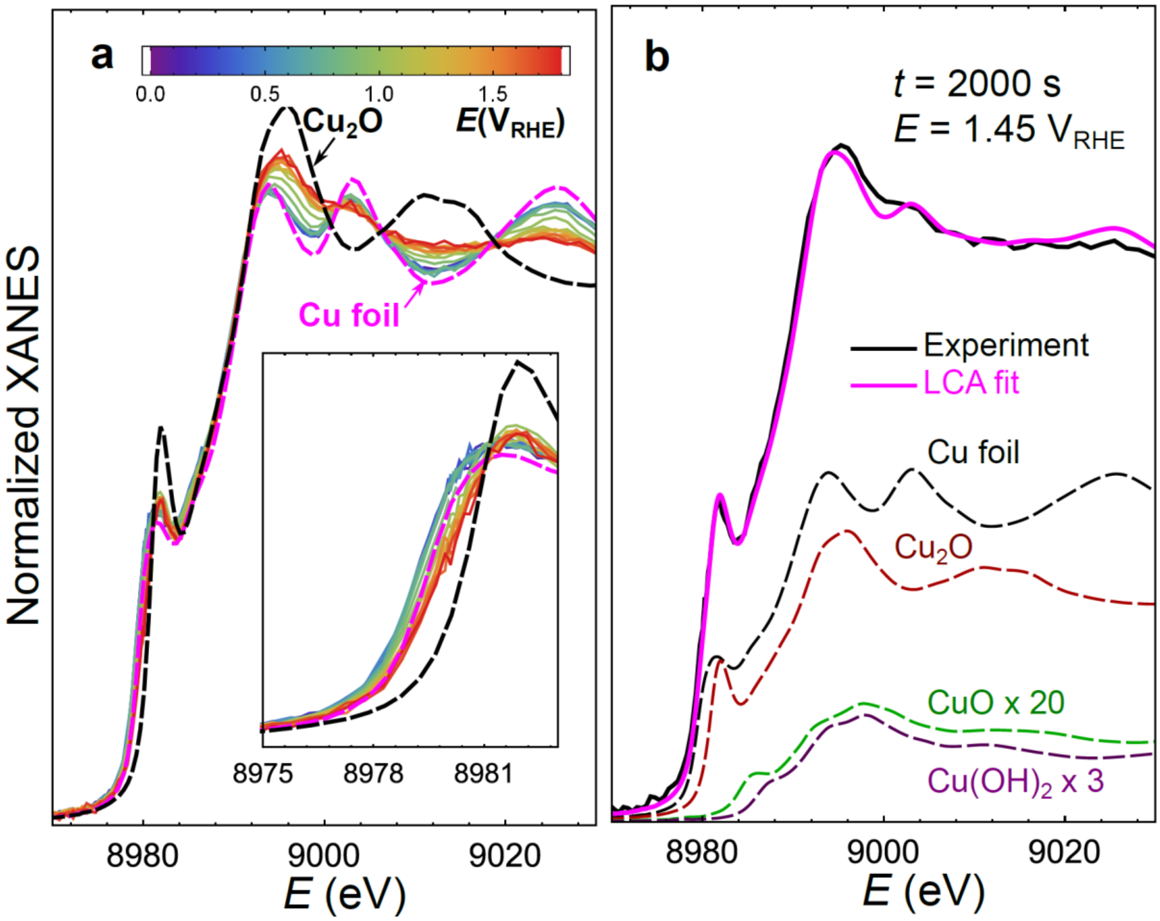

Figure S3. (a) Time-dependent Cu K-edge XANES data for Cu NCs acquired during the forward scan of the CV, when the potential is increased from 0 to +1.8 V *vs.* RHE (without IR correction). (b) LCA fit for the sample measured at +1.45 V *vs.* RHE. Dashed lines show the reference spectra used for LCA (XANES for metallic Cu, Cu_2O, CuO and $Cu(OH)_2$), scaled by their relative importance in the corresponding linear combination. For clarity, the scaled reference spectra for $Cu(OH)_2$ and CuO are further multiplied by factors 3 and 20, respectively.

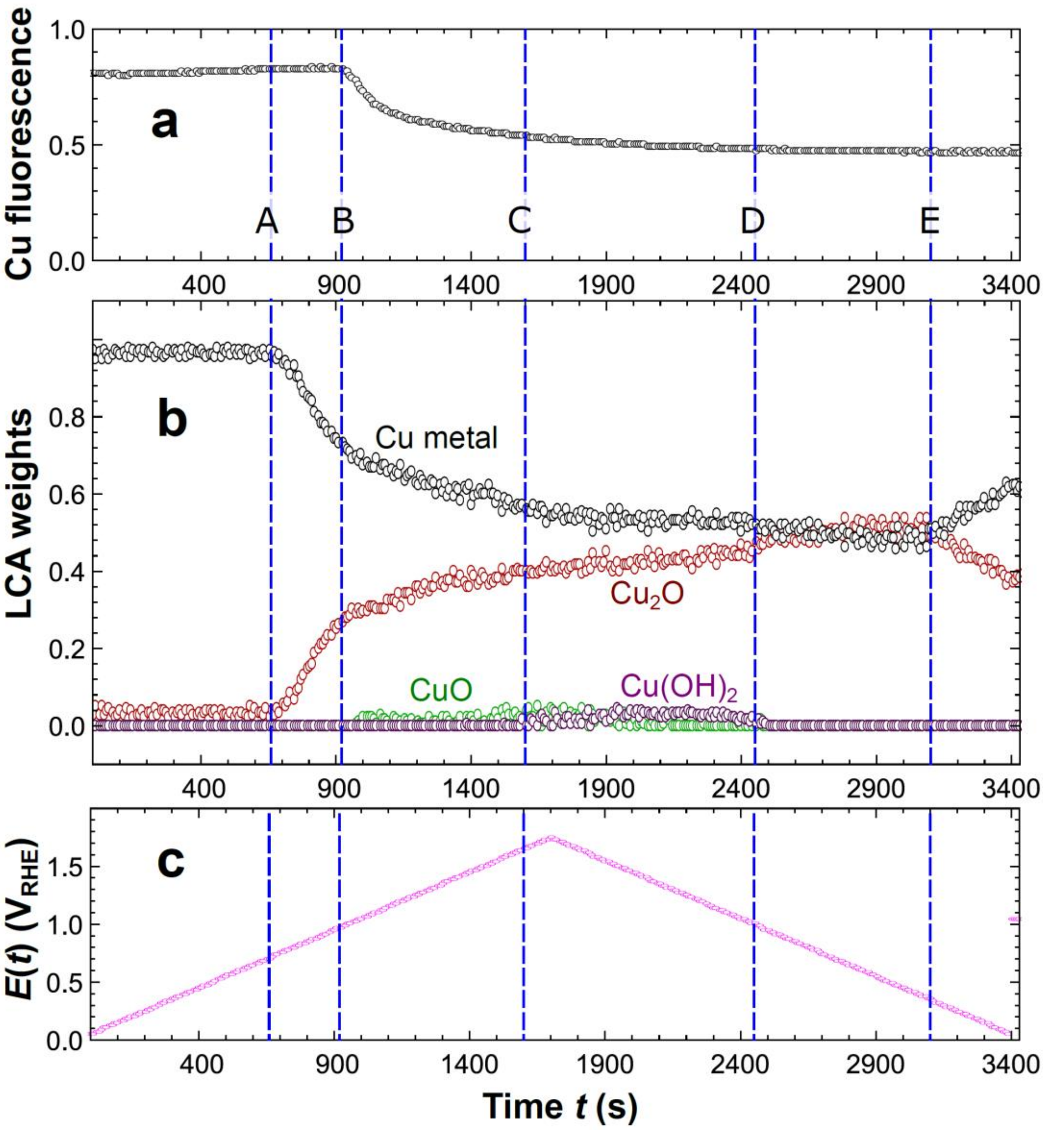

Figure S4. Oxidation and reduction of pre-reduced Cu NCs during a CV scan. (a) Changes in the fluorescence intensity of the Cu species during the CV scan. (b) Time-dependency of the LCA weights, corresponding to metallic Cu, Cu_2O, CuO and $Cu(OH)_2$ references, as obtained from time-resolved XANES data. (c) Corresponding changes in the applied potential (without IR correction). Vertical lines indicate the time moments, when we observe the onset of (A) Cu(I) species formation, (B) Cu(II) species formation, (C) formation of $Cu(OH)_2$-like species, (D) Cu(II) reduction, and (E) Cu(I) reduction.

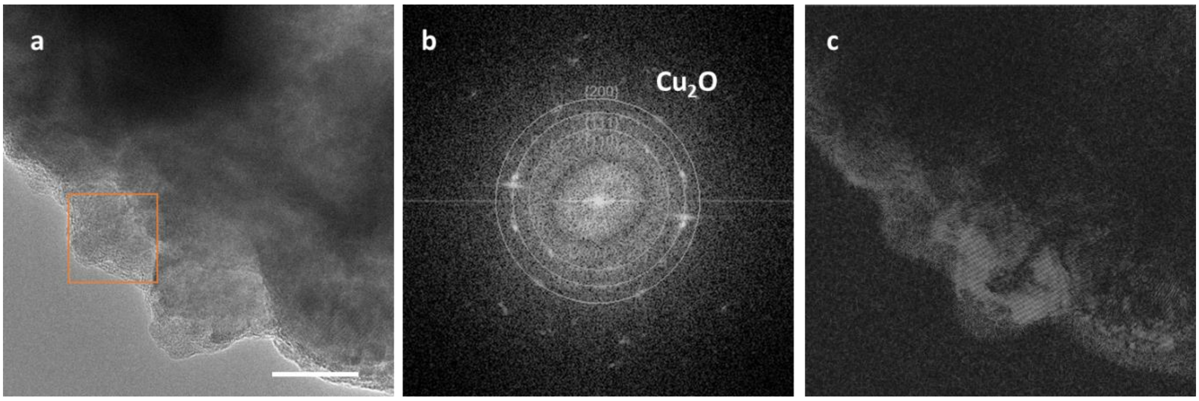

Figure S5. (a) TEM image and (b) corresponding fast Fourier transform (FFT, orange marked) from an oxide island at the edge of a Cu catalyst particle after pulsed electrolysis with EAn = 1.2 V, scale bar: 20 nm. (c) The inverse FFT image of the sample, where the FFT is selectively masked to retain only the {110} (0.30 nm) and {111} (0.24 nm) lattice spacings of Cu_2O. Since these fringes do not overlap with those for metallic Cu, the bright areas of the inverse images represent areas with surface oxide formed. It should be noted however that even though TEM reveals clearly the presence of Cu_2O species, the measurements were carried out *ex situ* and therefore, the oxidation state of our catalysts cannot be reliably concluded from these data but must be extracted from the *operando* XAS and SERS measurements.

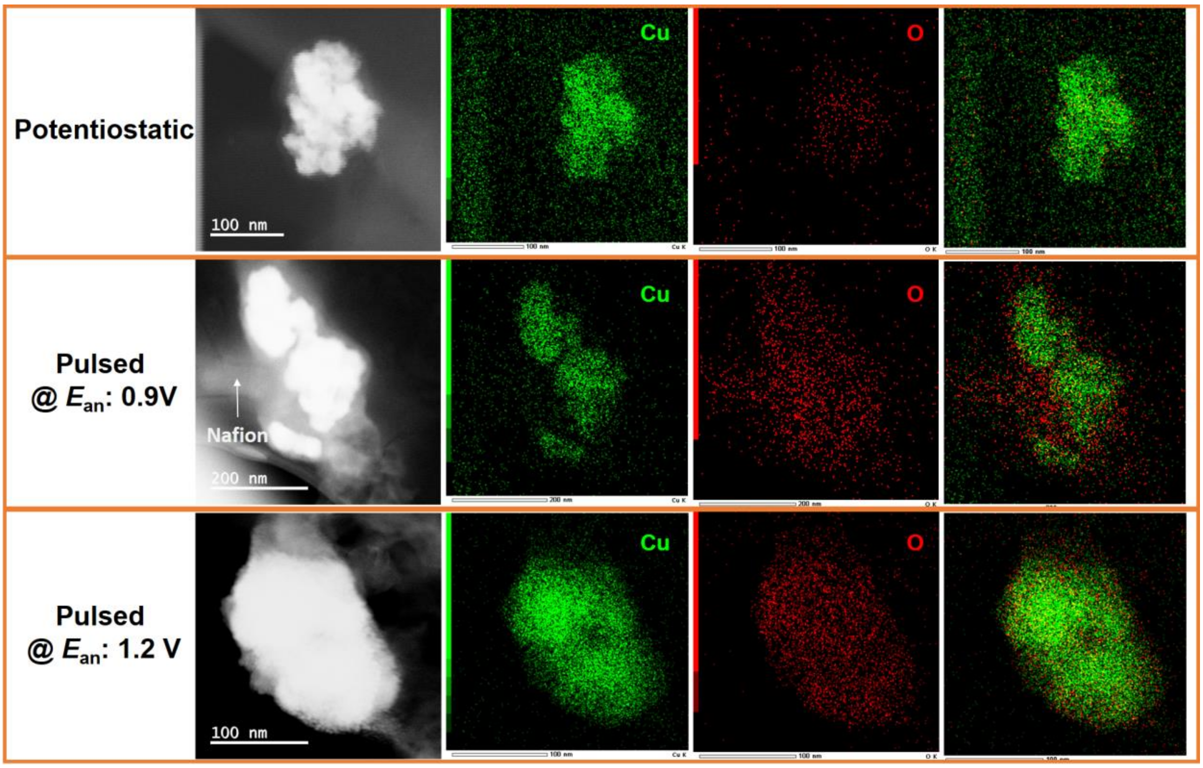

Figure S6. EDX-mapping analysis of Cu NCs samples after potentiostatic and after pulsed CO_2RR at E_{an} = 0.9 V and 1.2 V. Note that the EDX results for the sample exposed to pulsed CO_2RR at E_{an} = 0.9 V indicate that the residual Nafion binder also contributes to the oxygen signal in the maps. The latter makes difficult to conclude whether there is oxide formation in this sample under reaction conditions. Nevertheless, and despite the ex situ nature of the analysis, it is clear that the sample treated under potentiostatic CO_2RR is less oxidized than the samples exposed to pulsed CO_2RR.

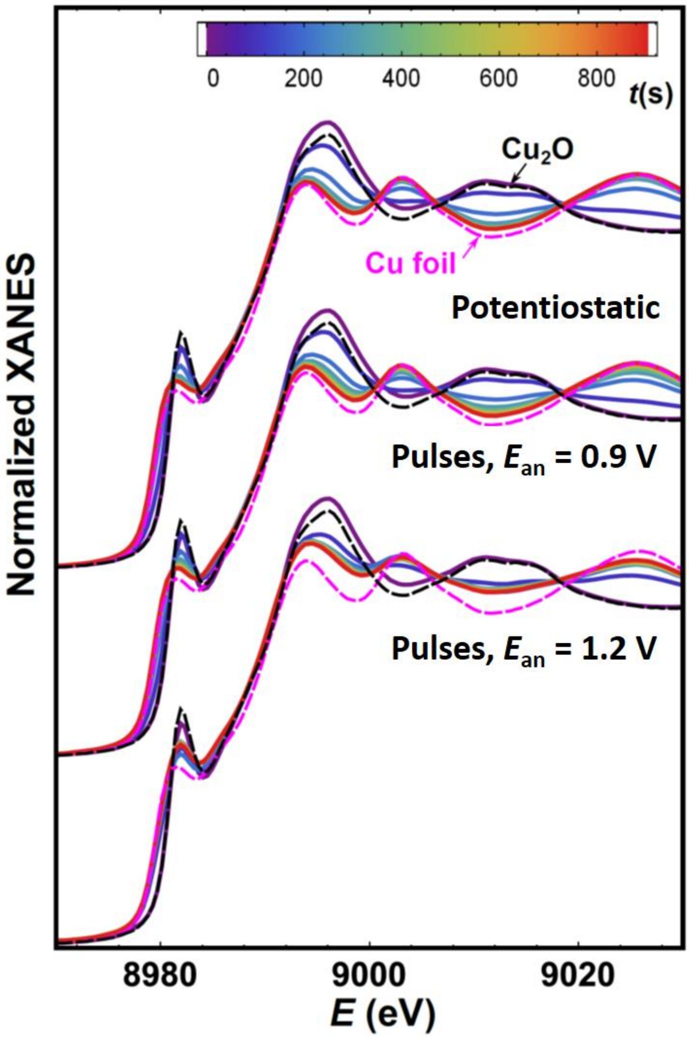

Figure S7 Time-dependent Cu K-edge XANES spectra for Cu NCs under potentiostatic CO_2RR and under pulsed reaction conditions with E_{an} = 0.9 and 1.2 V. Reference spectra for bulk Cu and Cu_2O standards are also shown. For clarity, spectra are shifted vertically.

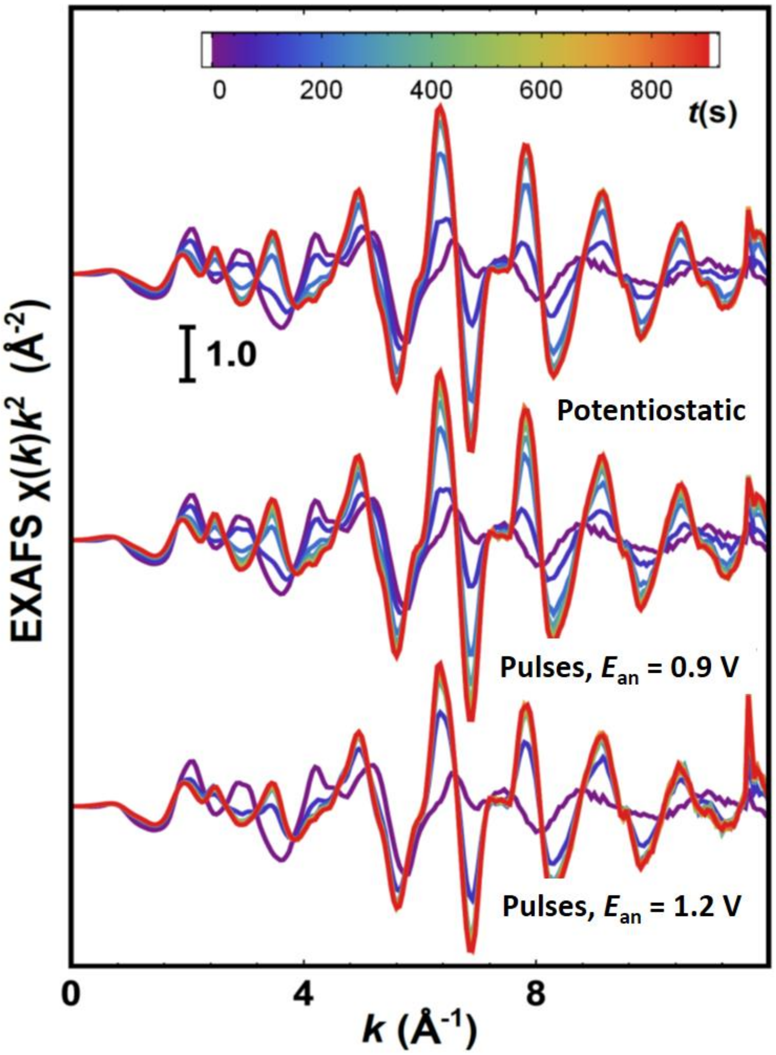

Figure S8. Time-dependent Cu K-edge EXAFS spectra for Cu NCs under potentiostatic CO_2RR and under pulsed reaction conditions with E_{an} = 0.9 and 1.2 V. For clarity, the spectra are shifted vertically.

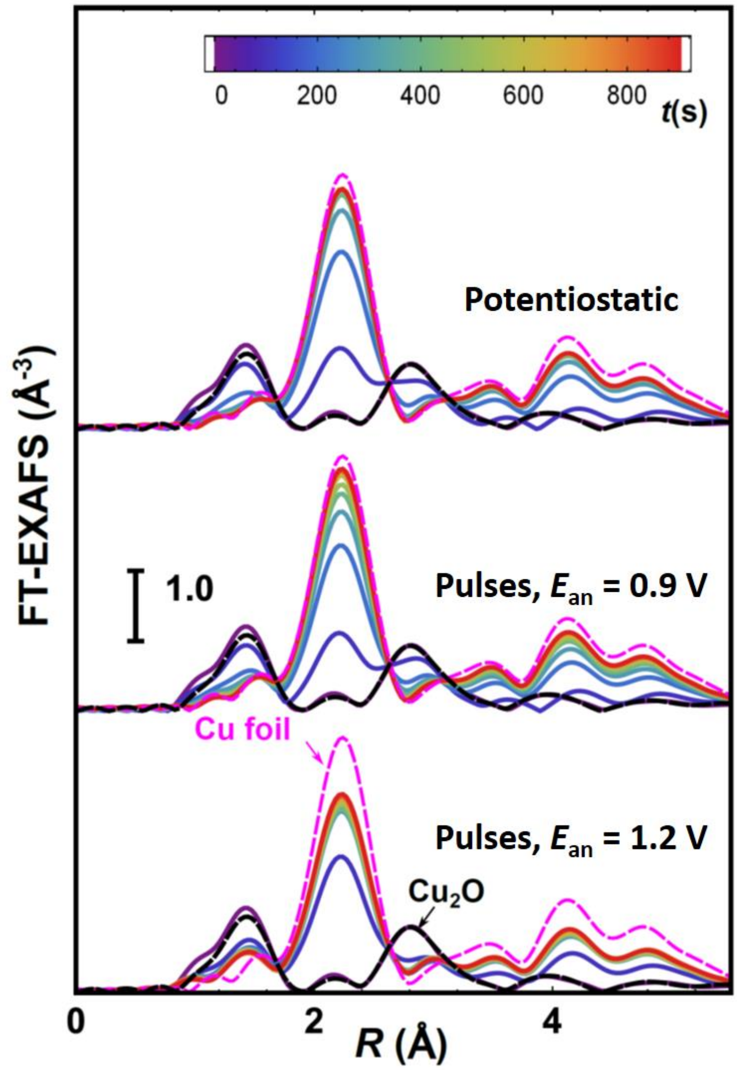

Figure S9. Time-dependent Fourier-transformed (FT) EXAFS spectra for Cu NCs under potentiostatic CO_2RR and under pulsed reaction conditions with E_{an} = 0.9 and 1.2 V. Reference spectra for bulk Cu and Cu_2O standards are also shown. For clarity, spectra are shifted vertically.

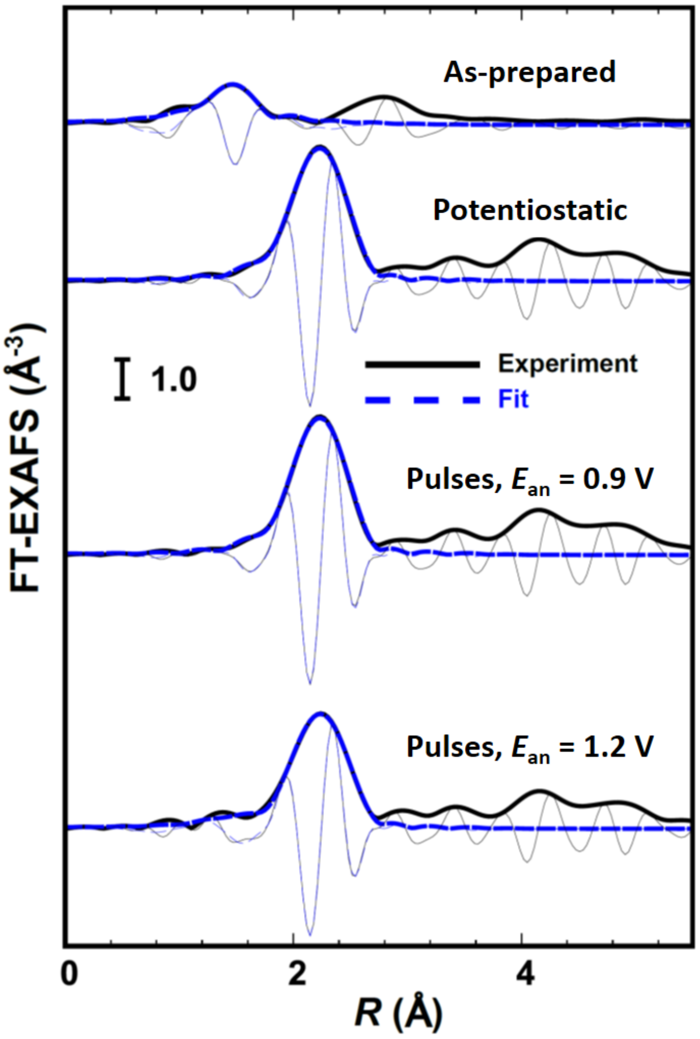

Figure S10. Representative examples of EXAFS fitting. Moduli (thick lines) and imaginary parts (thin lines) of Fourier-transformed experimental (black solid lines) and fitted (blue dashed lines) EXAFS spectra for as-prepared Cu NCs and for Cu NCs after 30 min under potentiostatic CO_2RR and after 30 min under pulsed reaction conditions with E_{an} = 0.9 and 1.2 V. Spectra are shifted vertically for clarity.

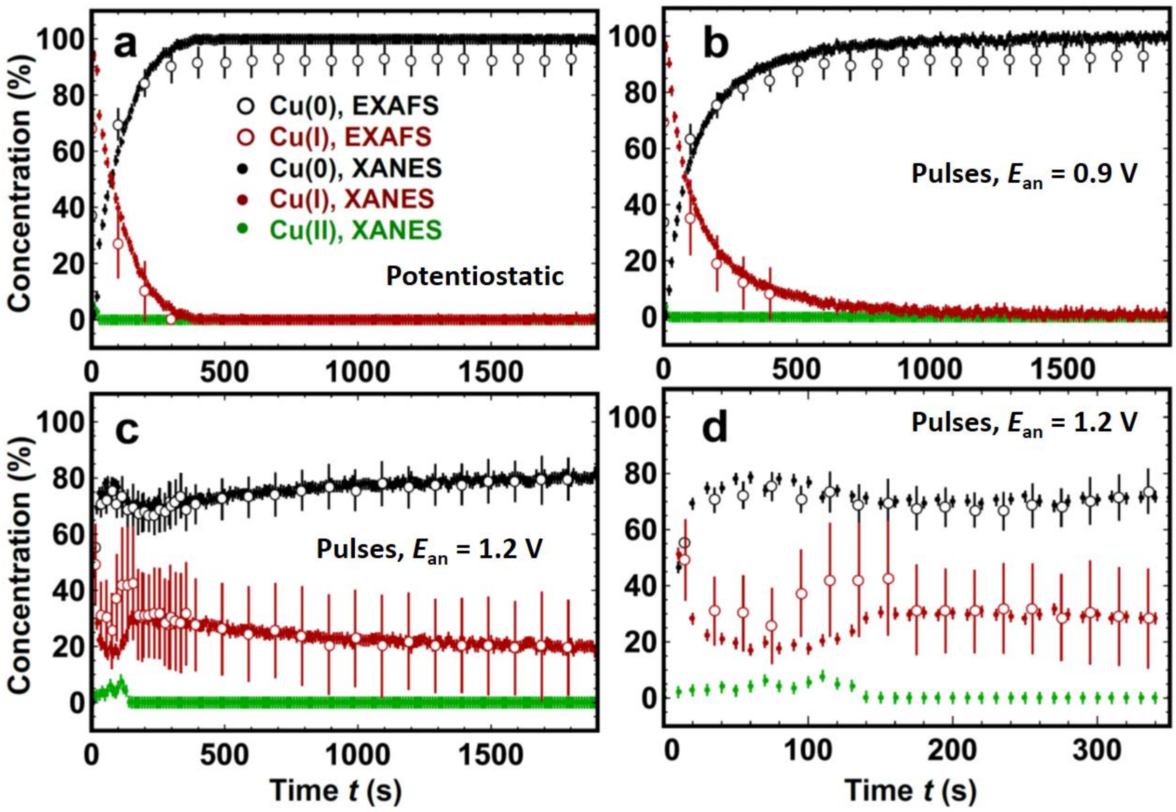

Figure S11. Concentrations of Cu(0), Cu(I) and Cu(II) species, as obtained from LCA-XANES results (filled circles) for (a) potentiostatic CO_2RR, and for pulsed CO_2RR with (b) E_{an} = 0.9 V and (c,d) 1.2 V. Spectra for metallic Cu, Cu_2O, CuO and $Cu(OH)_2$ were used as references for the LCA fitting. The Cu(II) concentration reported is the sum of CuO and $Cu(OH)_2$ contributions. Concentrations of Cu(0) and Cu(I), as estimated from the Cu-Cu and Cu-O coordination numbers ($N_{Cu\text{-}Cu}$ and $N_{Cu\text{-}O}$) obtained in EXAFS data fitting, are also shown (empty circles). The Cu(0) concentration is obtained as $N_{Cu\text{-}Cu}/12$, while the Cu-O concentration as $N_{Cu\text{-}O}/2$, where 12 and 2 are the numbers of nearest Cu neighbors in bulk metallic Cu and Cu_2O, respectively. For Cu(I), EXAFS and XANES results are expected to agree only when the concentration of 4-coordinated Cu(II) species can be neglected.

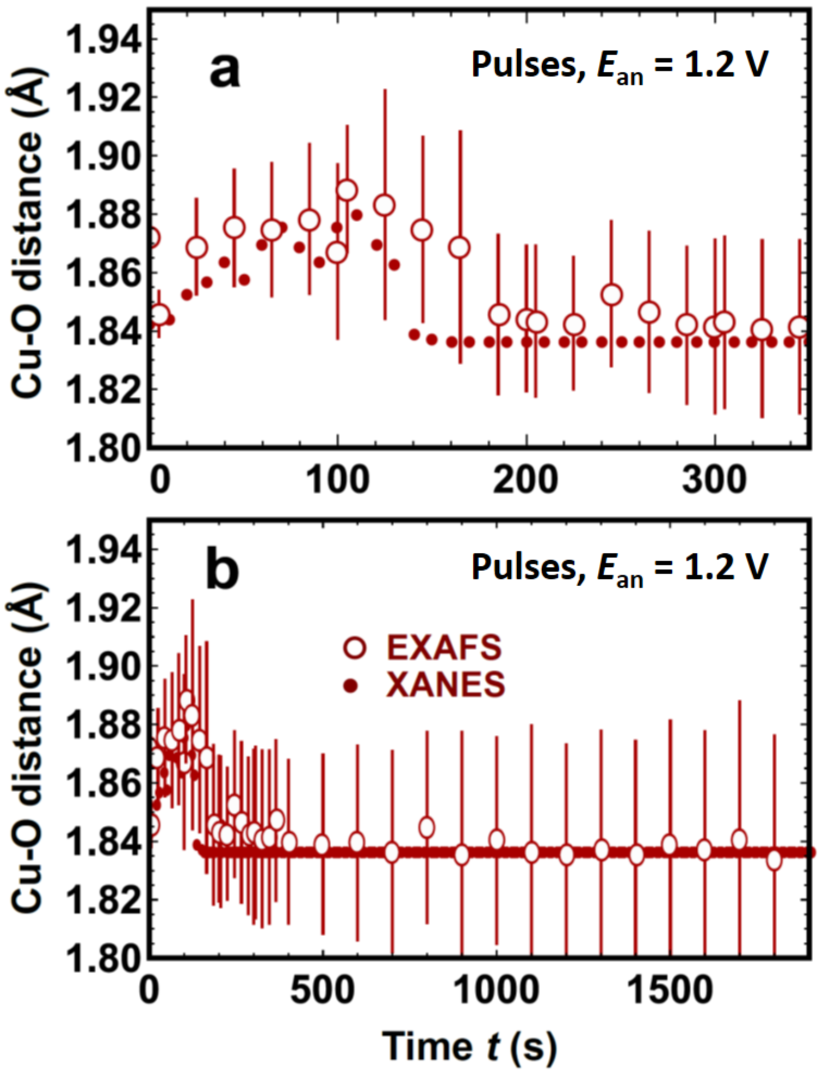

Figure S12. Time-dependency of the Cu-O interatomic distances within the first 350 s (a) and within the first 2000 s (b), as obtained from EXAFS data fitting for a sample exposed to pulsed CO_2RR with E_{an} = 1.2 V (empty circles). For comparison, the average Cu-O bond length, as estimated from LCA-XANES analysis is also shown (filled circles). The latter is obtained as $[2\times 1.836\ \text{Å}\times w_{Cu(I)} + 4\times 1.937\ \text{Å}\times w_{Cu(II)}]/ [2\times w_{Cu(I)} + 4\times w_{Cu(II)}]$, where $w_{Cu(I)}$ and $w_{Cu(II)}$ are the concentrations of Cu(I) and Cu(II) species, respectively, and 1.836 Å and 1.937 Å are Cu-O bond lengths in Cu_2O and CuO bulk oxides. Cu is 2-coordinated in Cu_2O, and 4-coordinated in CuO.

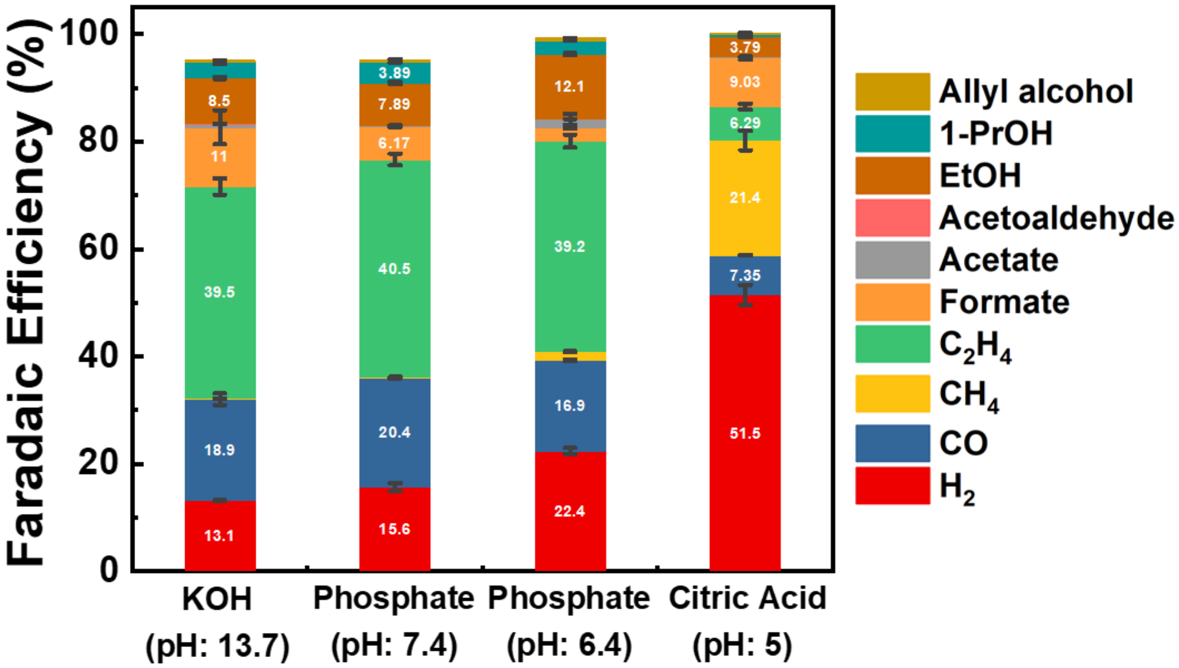

Figure S13. Faradaic efficiencies of Cu NCs in various electrolytes with different pH values. Note that since the difference in pH can cause a potential shift, here we used the chronopotentiometry technique (applied -200 mA/cm^2 for 30 min) for the comparison of the selectivity under the same reaction rate.

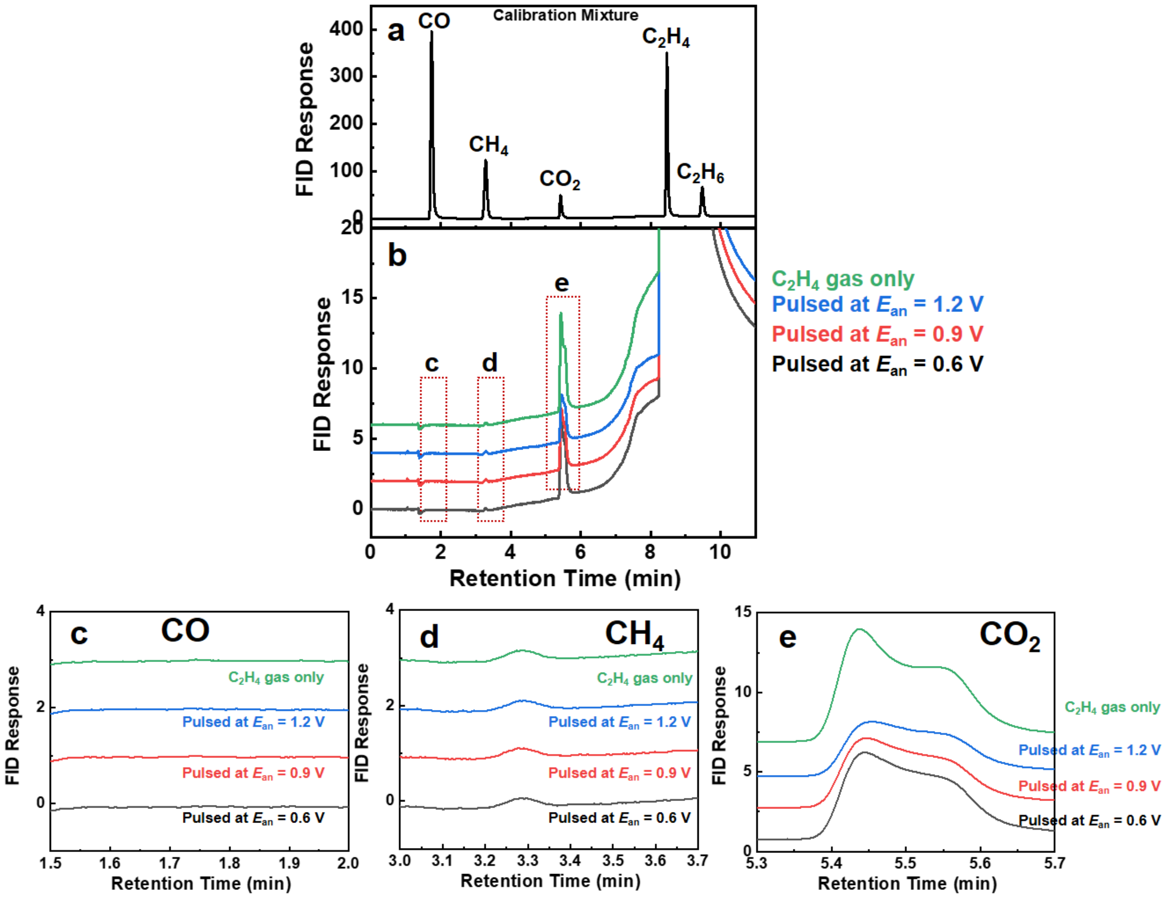

Figure S14 Gas chromatograms (GC-FID) recorded during the pulsed electrolysis with the different E_{an} values under C_2H_4 gas flow in 1 M KOH electrolyte. (a) Standard calibrated gas mixture of CO, CH_4, CO_2, C_2H_4, and C_2H_6 as references. (b) FID response under the pulsed electrolysis at E_{an} = 0.6, 0.9, and 1.2V, and C_2H_4 gas. (c-e) Enlarged chromatogram of Figure S14b for (c) CO, (d) CH_4, and (e) CO_2. Note here that the trance CH_4 and CO_2 signals detected in the FID stem from impurities in the C_2H_4 gas.

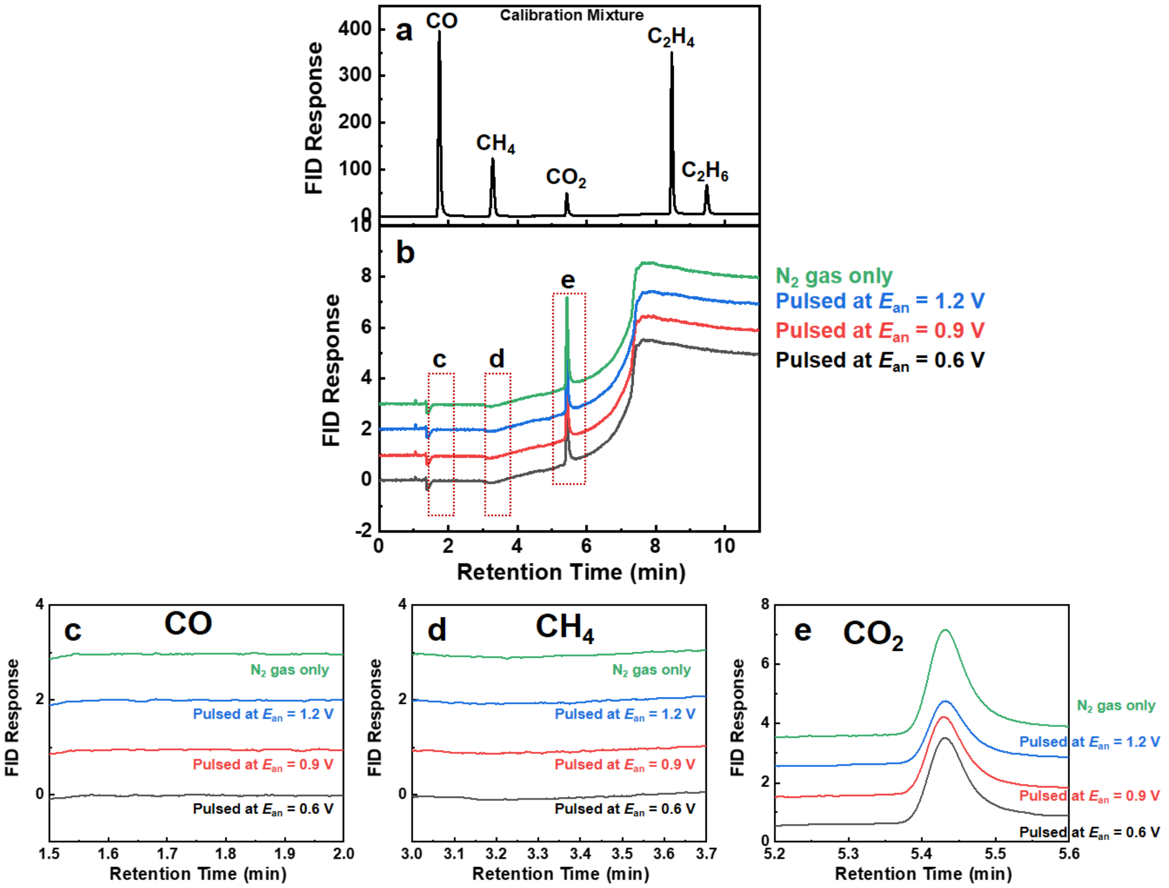

Figure S15 Gas chromatograms (GC-FID) recorded during the pulsed electrolysis with the different E_{an} values under N_2 gas flow in 1 M KOH electrolyte including 10 mM EtOH. (a) Standard calibrated gas mixture of CO, CH_4, CO_2, C_2H_4, and C_2H_6 as references. (b) FID response under the pulsed electrolysis at E_{an} = 0.6, 0.9, and 1.2 V, and N_2 gas. (c-e) Enlarged chromatogram of Figure S15b for (c) CO, (d) CH_4, and (e) CO_2. Note here that the trance CO_2 amount detected in the FID stems from the impurity of the N_2 gas.

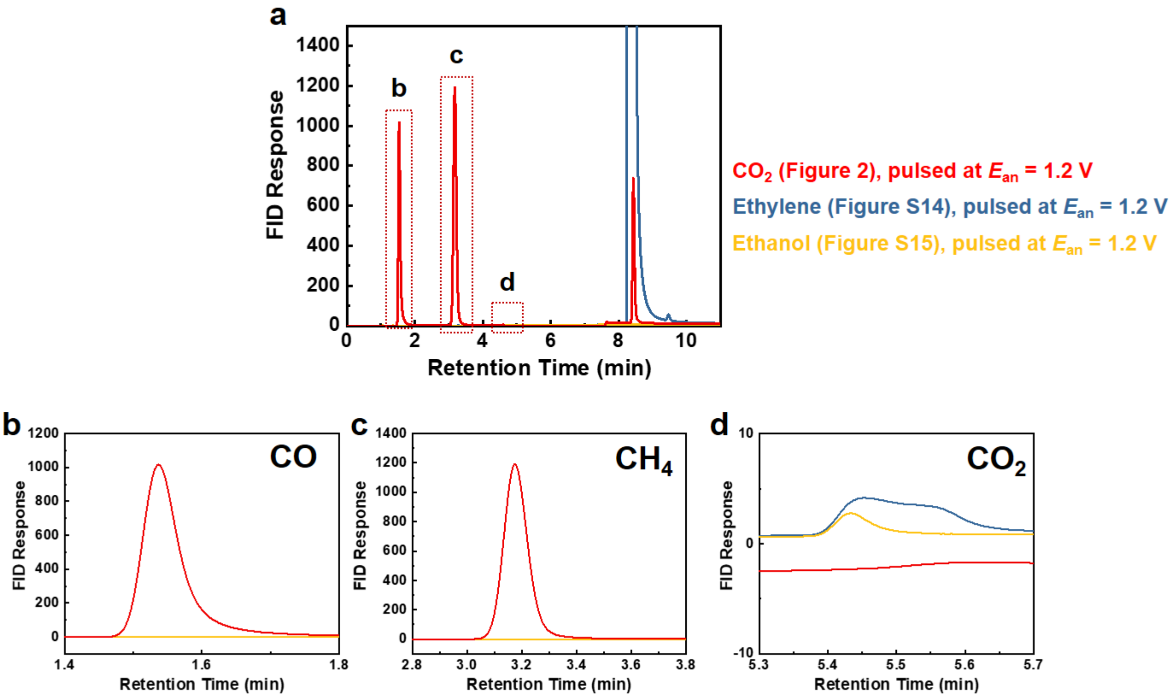

Figure S16. Data comparison of gas chromatograms (GC-FID) recorded during the pulsed electrolysis at the E_{an} = 1.2 V under CO_2 gas flow in 1 M KOH (Red, Figure 2), ethylene gas flow in 1 M KOH (Blue, Figure S14), and N_2 gas flow in 1 M KOH including 10 mM EtOH (Yellow, Figure S15). (b-d) enlarged chromatogram of Figure S16a for (b) CO, (c) CH_4, and (d) CO_2. Note here that no CO_2 gas is detected under CO_2 gas flow conditions (red) because CO_2 gas is released through the GC's bypass valve. High concentration of CO_2 gas can strain the methanizer accessory.

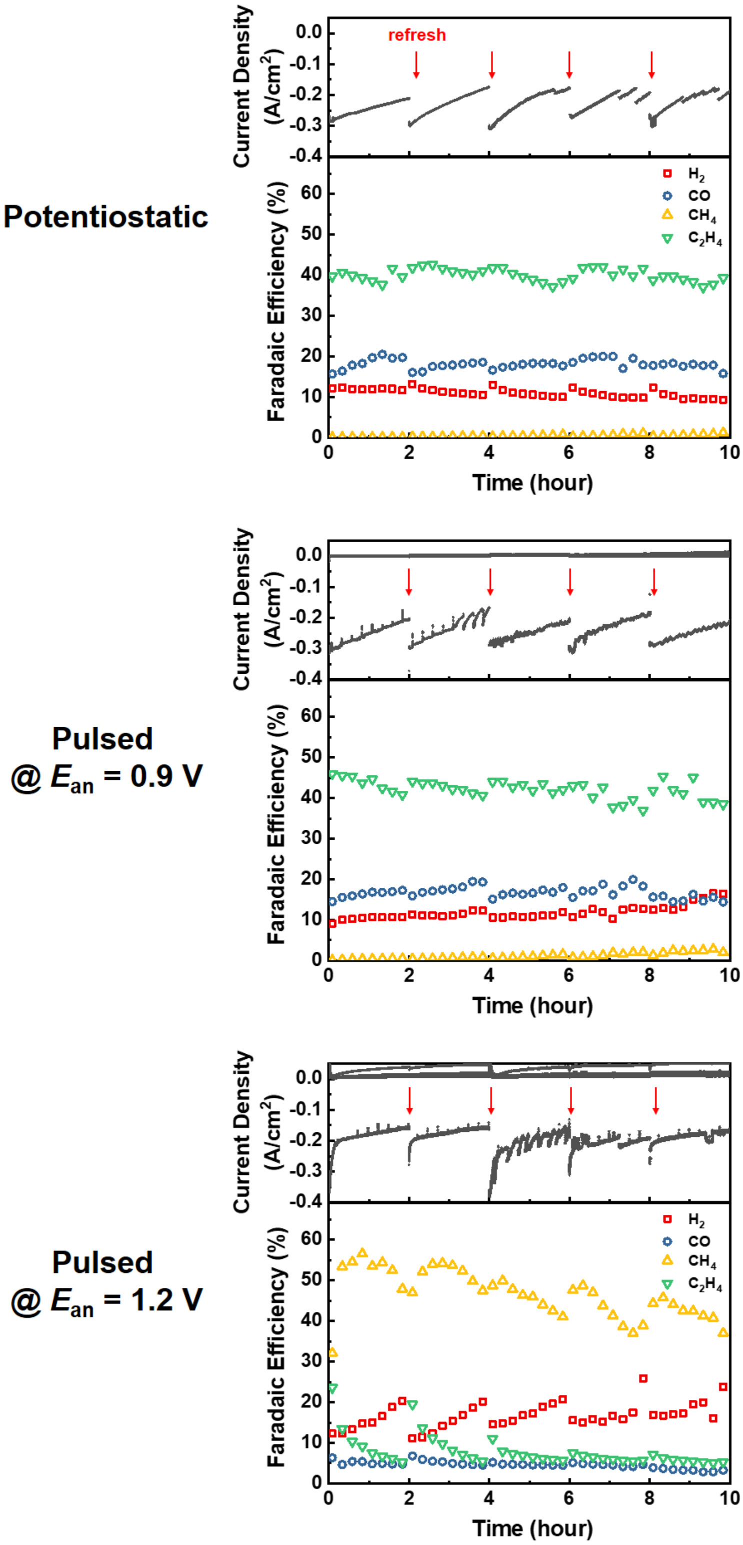

Figure S17. Current density (top) and Faraday efficiency (bottom) of gas products obtained under potentiostatic CO_2RR conditions at -0.7 V *vs.* RHE and during the pulsed electrolysis with E_{an} = 0.9 V and 1.2 V for 10 hours. The red arrows indicate the times at which the electrolyte was refreshed.

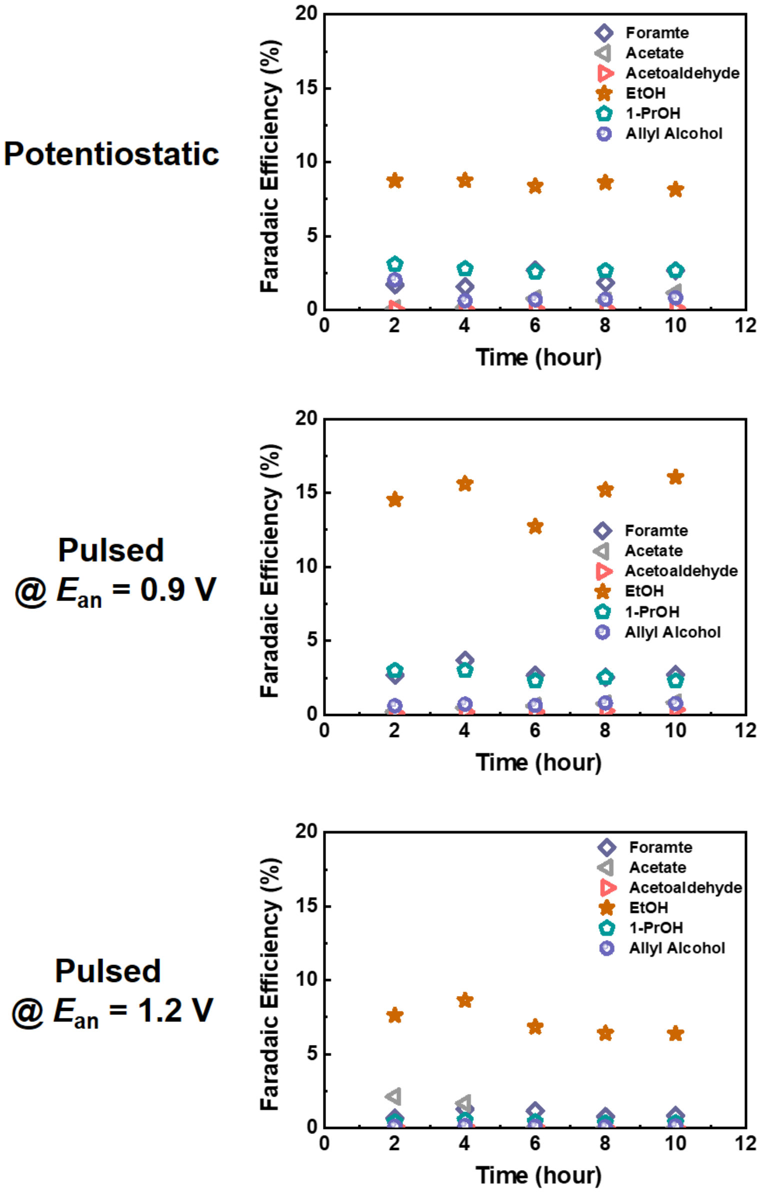

Figure S18. Faraday efficiency of liquid products obtained under potentiostatic CO_2RR conditions at -0.7 V *vs.* RHE and during pulsed electrolysis with E_{an} = 0.9 V and 1.2 V for 10 hours.

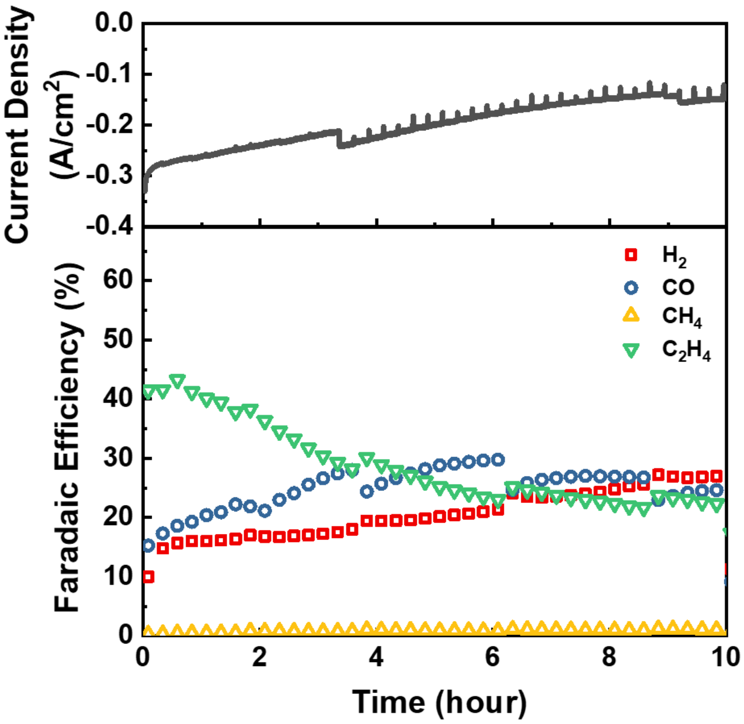

Figure S19. Current density (top) and FE (bottom) at potentiostatic CO_2RR conditions at -0.7 V *vs.* RHE for 10 hours. Here, unlike in Figure S14, the electrolyte is not refreshed periodically, which causes a gradual change in the selectivity and a decrease in current.

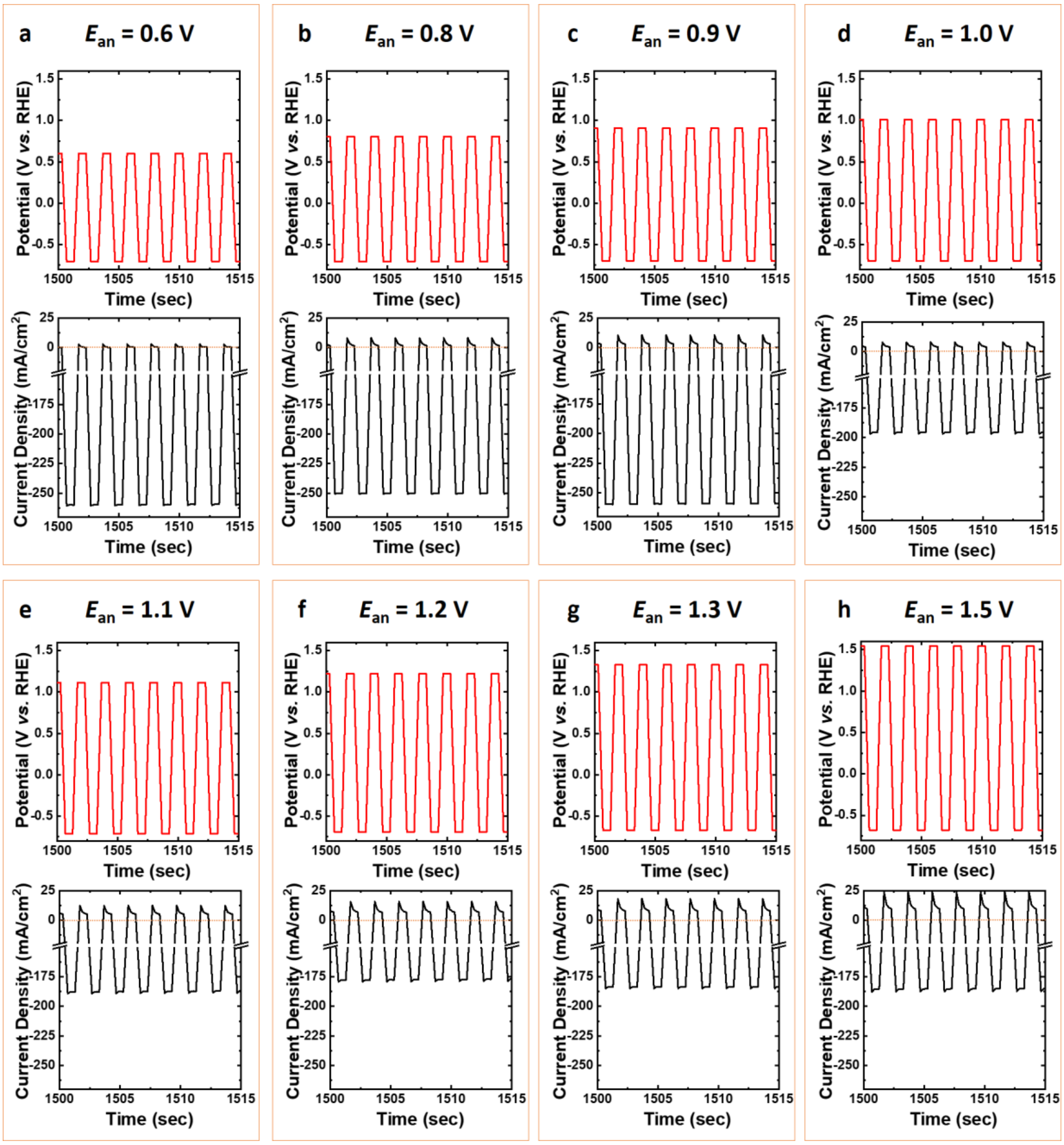

Figure S20. Potential and current profiles of Cu NCs during pulsed electrolysis with the different E_{an} values indicated and the same E_{ca} = -0.7 V vs. RHE cathodic potential in all cases.

Supplementary References

S1. Bornmann, B.; Kläs J.; Müller O.; Lützenkirchen-Hecht D.; Frahm R., The quick EXAFS setup at beamline P64 at PETRA III for up to 200 spectra per second. *AIP Conf. Proc.* **2019,** 2054, 040008.

S2. Ravel, B.; Newville, M., ATHENA, ARTEMIS, HEPHAESTUS: data analysis for X-ray absorption spectroscopy using IFEFFIT. *J. Synchrotron Radiat.* **2005,** *12*, 537-541.

S3. Ankudinov, A. L.; Ravel, B.; Rehr, J. J.; Conradson, S. D., Real-space multiple-scattering calculation and interpretation of x-ray-absorption near-edge structure. *Phys. Rev. B* **1998,** *58*, 7565-7576.
